# Supplementary material for: Isomer design unlocks rainbow phosphorescence
Source: Nat Commun. 2026 Mar 17;17:4093. doi: 10.1038/s41467-026-70784-7 (PMC13144461; doi:10.1038/s41467-026-70784-7)
Supplement: Supplementary file 1 — Supplementary Information [file 41467_2026_70784_MOESM1_ESM.pdf]

Supplementary Information

Isomer design unlocks rainbow phosphorescence

Xinyue Xu[1], Dong Ding[1], Xinyu Ding[1], Shaoyang Han[1], Erkin Zakhidov[2], Feng Li[3*], Mingliang Sun[1*]

[1]School of Materials Science and Engineering, Ocean University of China, Qingdao, 266100, China.

[2]Institute of Ion-Plasma and Laser Technologies of the Academy of Sciences of the Republic of Uzbekistan, Tashkent 100125, Uzbekistan.

[3]Analytical and Testing Center, Qingdao University of Science & Technology, Qingdao 266042, China.

*Correspondence to: mlsun@ouc.edu.cn; lifeng02@qust.edu.cn.

Syntheses and characterizations

The carbazole (Cz) configuration was synthesized using the solution method in the following steps[1].

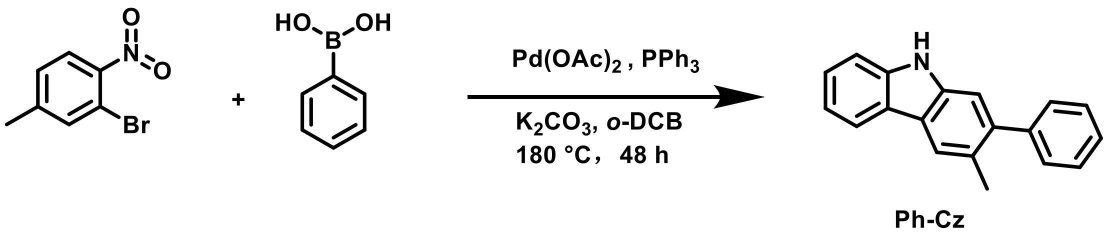

Supplementary Figure. 1 The synthetic route to **Ph-Cz**.

Add 2-bromo-4-methyl-1-nitrobenzene (0.432 g, 2 mmol), phenylboronic acid (0.586 g, 4.8 mmol), K_2CO_3 (1.104 g, 8 mmol), palladium acetate (45 mg, 0.2 mmol) and PPh3 (2.623 g, 10 mmol) to a 50 ml reaction vial and reflux under nitrogen for 48 hours in 15 ml o-dichloroethane solvent at a temperature of 180 °C. for 48 hours in 15 ml of o-dichloroethane solvent under nitrogen at 180 °C. After the reaction was cooled to room temperature, it was extracted with water and dichloromethane, and the organic phase was purified on a silica gel column using petroleum ether-ethyl acetate 5:1 as

eluent to give the light brown product Ph-Cz. Yield: 70%. ^{1}H NMR (500 MHz, DMSO-d_6) δ 11.10 (s, 1H), 8.06 (d, J = 7.7 Hz, 1H), 7.97 (s, 1H), 7.43 (t, J = 7.2 Hz, 3H), 7.38 (d, J = 7.3 Hz, 2H), 7.37 – 7.30 (m, 2H), 7.25 (s, 1H), 7.11 (t, J = 7.4 Hz, 1H), 2.32 (s, 3H).

^{13}C NMR (126 MHz, DMSO-d_6) δ 142.44, 140.30, 139.39, 138.41, 129.22, 128.17, 126.70, 125.42, 124.92, 122.10, 121.83, 121.24, 120.14, 118.45, 111.66, 110.93, 20.56. HR-MS Calcd. For $C_{19}H_{15}N$ $[M-H]^+$: 258.1277. Found: 258.1241.

The synthesis of benzindole Bd[g] and Bd[e] configuration was carried out by ball milling method in the following steps[2].

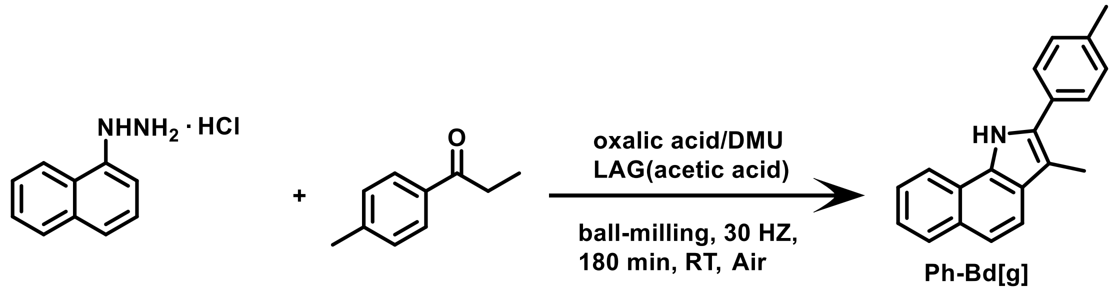

Supplementary Figure. 2 The synthetic route to **Ph-Bd[g]**.

Add 1-naphthylhydrazine hydrochloride (0.390 g, 2 mmol), 1-(p-tolyl)propan-1-one (0.326 g, 2.2 mmol), oxalic acid (0.64 g, 7 mmol), dimethylurea (0.28 g, 3 mmol), and 40 microliters of acetic acid to a 15-ml ball mill jar. Two small steel balls of 10 mm diameter were then added to the jar. The reaction jar was fixed on a ball mill and the frequency of the ball mill was set to 30 HZ. The product was ground in air at room temperature for 3 h. The product was cooled to room temperature, then washed with water and filtered to obtain a solid crude product. The crude product was purified by silica gel column with petroleum ether-ethyl acetate 5:1 as eluent to obtain the light pink pure product. Yield: 76%. ^{1}H NMR (500 MHz, Chloroform-d) δ 8.70 (s, 1H), 8.03 (d, J = 8.1 Hz, 1H), 7.96 (d, J = 8.0 Hz, 1H), 7.72 (d, J = 8.6 Hz, 1H), 7.55 (q, J = 10.6, 9.3 Hz, 4H), 7.44 (t, J = 7.5 Hz, 1H), 7.34 (d, J = 7.7 Hz, 2H), 2.55 (s, 3H), 2.46 (s, 3H).

^{13}C NMR (126 MHz, Chloroform-*d*) δ 137.00, 132.71, 130.64, 130.62, 130.21, 129.62, 129.01, 127.56, 125.78, 125.40, 123.76, 121.52, 120.37, 119.43, 119.01, 110.03, 21.25, 9.81.

HR-MS Calcd. For $C_{20}H_{17}N$ $[M-H]^+$: 272.1434. Found: 272.1428.

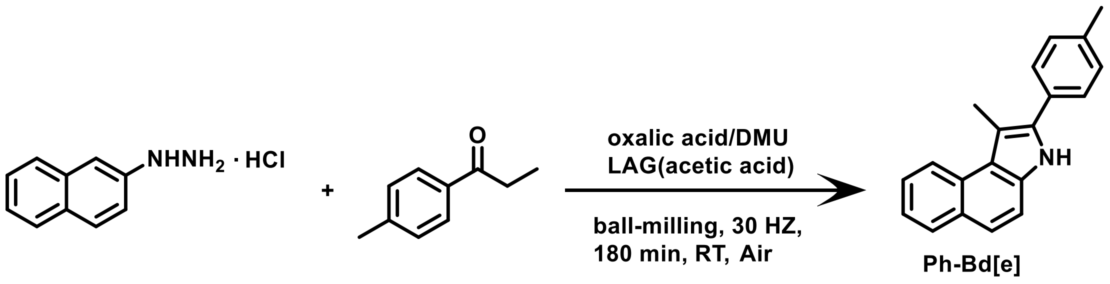

Supplementary Figure. 3 The synthetic route to **Ph-Bd[e]**.

Add 2-naphthylhydrazine hydrochloride (0.390 g, 2 mmol), 1-(p-tolyl)propan-1-one (0.326 g, 2.2 mmol), oxalic acid (0.64 g, 7 mmol), dimethylurea (0.28 g, 3 mmol), and 40 microliters of acetic acid to a 15-ml ball mill jar. Two small steel balls of 10 mm diameter were then added to the jar. The reaction jar was fixed on a ball mill and the frequency of the ball mill was set to 30 HZ. The product was ground in air at room temperature for 3 h. The product was cooled to room temperature, then washed with water and filtered to obtain a solid crude product. The crude product was purified by silica gel column with petroleum ether-ethyl acetate 5:1 as eluent to obtain the light pink pure product. Yield: 46%. ^{1}H NMR (500 MHz, Chloroform-*d*) δ 8.59 (d, *J* = 8.3 Hz, 1H), 8.31 (s, 1H), 7.94 (d, *J* = 8.0 Hz, 1H), 7.63 – 7.55 (m, 2H), 7.50 (dd, *J* = 14.1, 8.3 Hz, 3H), 7.44 (t, *J* = 7.4 Hz, 1H), 7.32 (d, *J* = 7.7 Hz, 2H), 2.83 (s, 3H), 2.45 (s, 3H).

^{13}C NMR (126 MHz, Chloroform-*d*) δ 137.21, 133.44, 132.40, 130.40, 129.95, 129.72, 129.54, 128.89, 128.45, 125.56, 123.30, 123.27, 122.84, 122.40, 112.55, 110.89, 21.26, 13.30.

HR-MS Calcd. For $C_{20}H_{17}N$ $[M-H]^+$: 272.1434. Found: 272.1420.

The benzindole [f] configuration was synthesized using a solution method in the following steps[3-7].

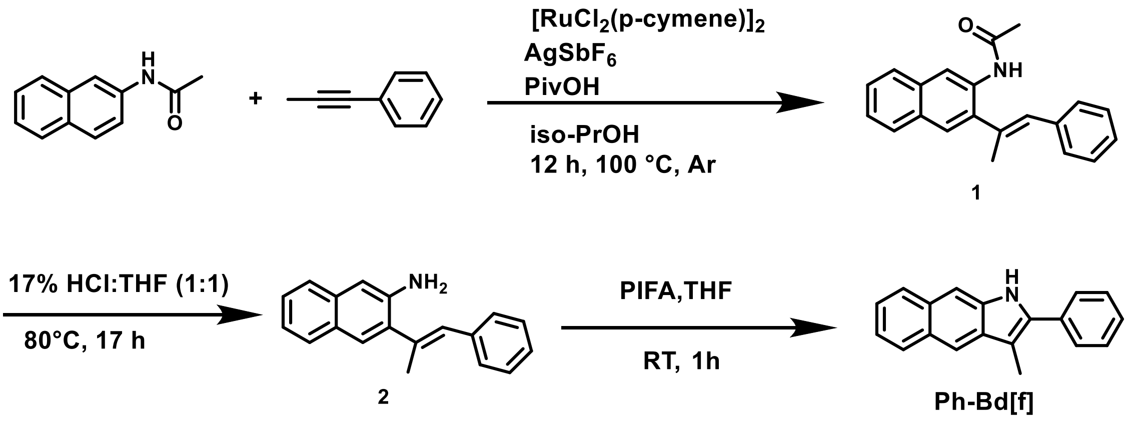

Supplementary Figure. 4 The synthetic route to **Ph-Bd[f]**.

N-(naphthalen-2-yl)acetamide (0.370 g, 2 mmol), [{RuCl2(pcymene)}2] (0.01 g, 5.0 mol%) and $AgSbF_6$ (0.01 g, 20 mol%) were pumped under argon gas for three times in a 25 ml reaction jar. Then 1-propyn-1phenyl (0.28 ml, 2.2 mmol), pivalic acid (0.5 ml, 5 mmol) and 10 ml of isopropanol solvent were added in an argon atmosphere, and then the reaction was refluxed at 100 °C for 12 h. The reaction was cooled down to room temperature, and then extracted with water and dichloromethane, and the organic phase was purified by silica gel column with petroleum ether-ethyl acetate 3:1 as eluent to give white intermediate 1 Pure product. Yield: 90%. ^{1}H NMR (500 MHz, Chloroform-*d*) δ 8.78 (s, 1H), 7.85 (d, *J* = 8.0 Hz, 1H), 7.76 (d, *J* = 7.5 Hz, 1H), 7.69 (s, 1H), 7.56 (s, 1H), 7.45 (d, *J* = 11.4 Hz, 6H), 7.33 (s, 1H), 6.64 (s, 1H), 2.32 (s, 3H), 2.20 (s, 3H).

The reaction was then carried out at 100°C for 17 h in the presence of a 1:1 mixture of 17% hydrochloric acid and tetrahydrofuran, and intermediate 1 was converted to intermediate 2 in 91% yield. ^{1}H NMR (500 MHz, Chloroform-*d*) δ 7.74 (d, *J* = 8.1 Hz, 1H), 7.64 (d, *J* = 6.9 Hz, 2H), 7.44 (ddd, *J* = 19.1, 12.7, 7.5 Hz, 5H), 7.29 (t, *J* = 8.8 Hz, 2H), 7.10 (s, 1H), 6.69 (s, 1H), 4.18 (s, 2H), 2.36 (s, 3H).

^{13}C NMR (126 MHz, Chloroform-*d*) δ 137.55, 136.47, 134.80, 134.13, 130.51, 129.04, 128.33, 128.02, 127.59, 127.53, 126.83, 126.10, 125.43, 122.68, 109.25, 19.47.

A solution of bis(trifluoroacetoxy)iodine (PIFA) (400 mg, 0.93 mmol) in tetrahydrofuran (3 mL) was added dropwise (10 min) to a solution of intermediate 2 (200 mg, 0.77 mmol) in tetrahydrofuran (3 mL) under argon. The resulting mixture was

stirred for another 1 h at room temperature, filtered through a short pad of silica gel and concentrated under reduced pressure to give the benzindole [f] product. The light yellow solid product Ph-Bd[f] was obtained in 20% yield using hexane-dichloromethane 4:1 as eluent. ^{1}H NMR (500 MHz, Chloroform-*d*) δ 8.06 (s, 1H), 7.98 (d, J = 7.8 Hz, 1H), 7.89 (d, J = 8.2 Hz, 2H), 7.77 (s, 1H), 7.67 (d, J = 7.4 Hz, 2H), 7.52 (t, J = 7.5 Hz, 2H), 7.37 (dq, J = 23.6, 7.0 Hz, 3H), 2.57 (s, 3H).

HR-MS Calcd. For $C_{19}H_{15}N$ $[M-H]^+$: 258.1277. Found: 258.1254.

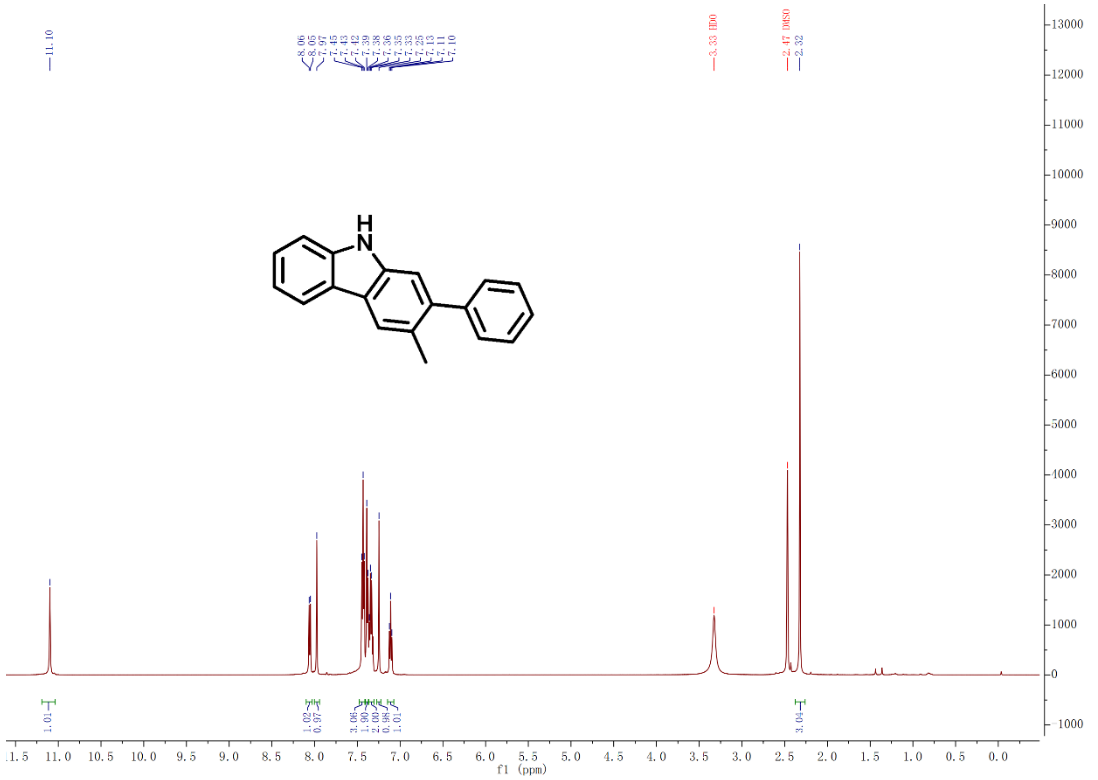

Supplementary Figure. 5 ^{1}H NMR spectrum of Ph-Cz in DMSO-d_6. (500 MHz, DMSO-d_6)

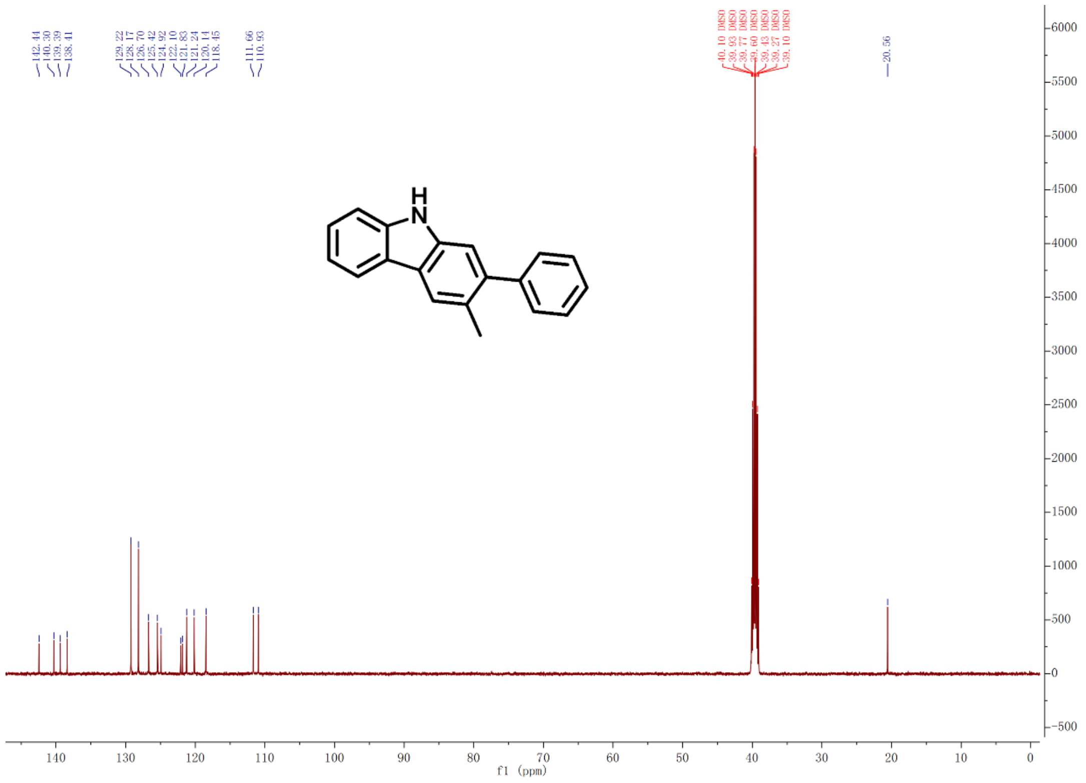

Supplementary Figure. 6 ^{13}C NMR spectrum of Ph-Cz in DMSO-d_6. (126 MHz, DMSO-d_6)

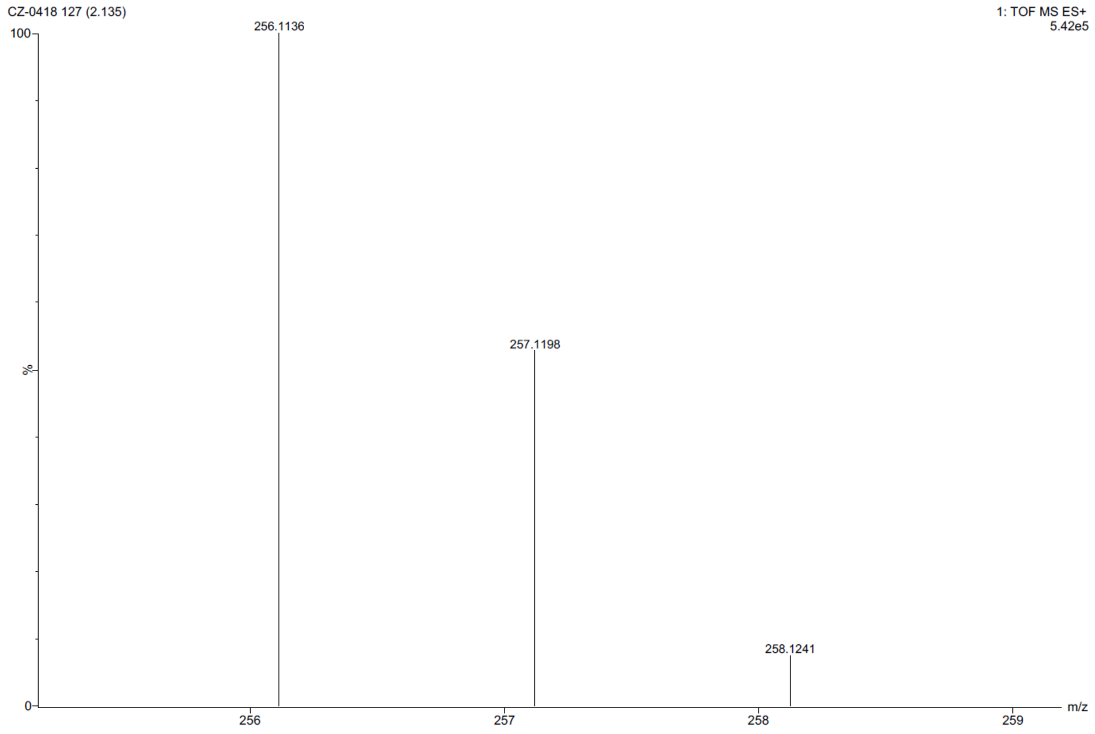

Supplementary Figure. 7 HR-MS spectrum of Ph-Cz.

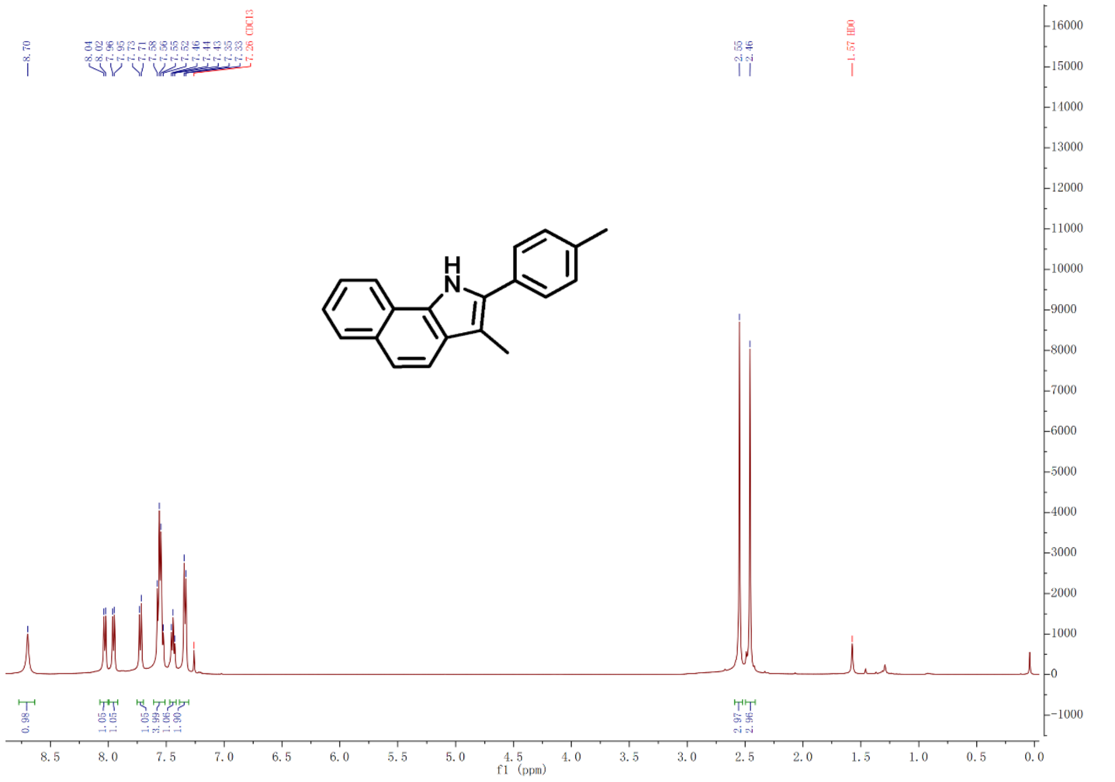

Supplementary Figure. 8 ^{1}H NMR spectrum of Ph-Bd[g] in $CDCl_3$. (500 MHz, Chloroform-*d*)

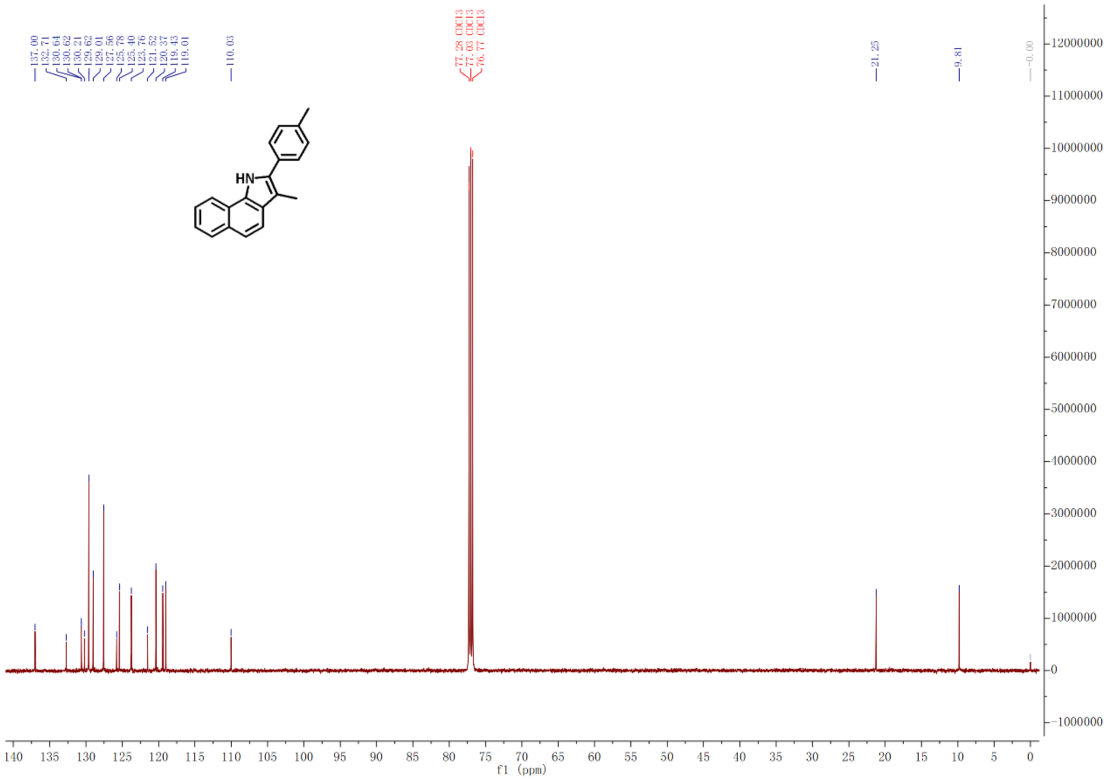

Supplementary Figure. 9 ^{13}C NMR spectrum of Ph-Bd[g] in $CDCl_3$. (126 MHz, Chloroform-*d*)

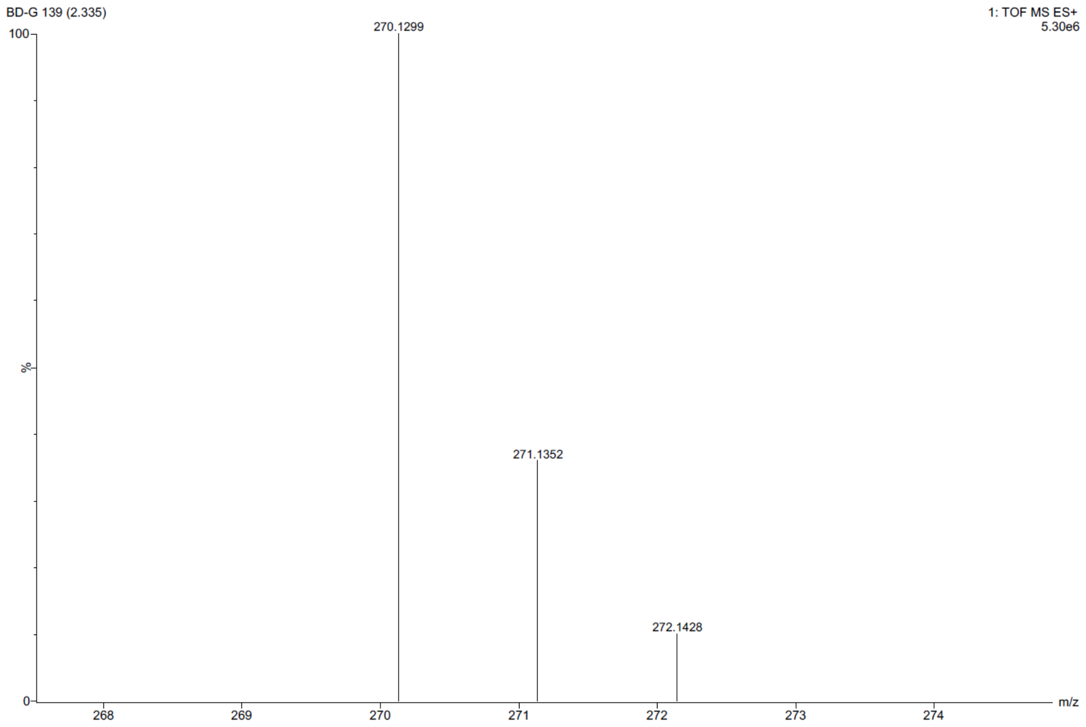

Supplementary Figure. 10 HR-MS spectrum of Ph-Bd[g].

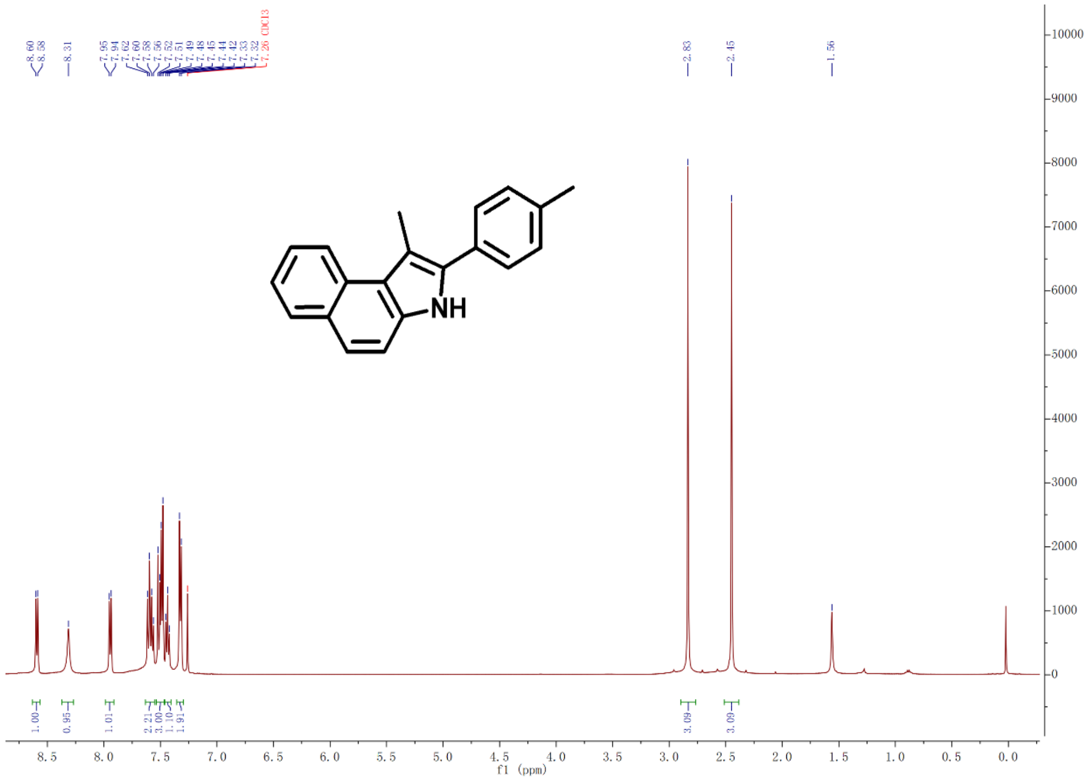

Supplementary Figure. 11 ^{1}H NMR spectrum of Ph-Bd[e] in $CDCl_3$. (500 MHz, Chloroform-*d*)

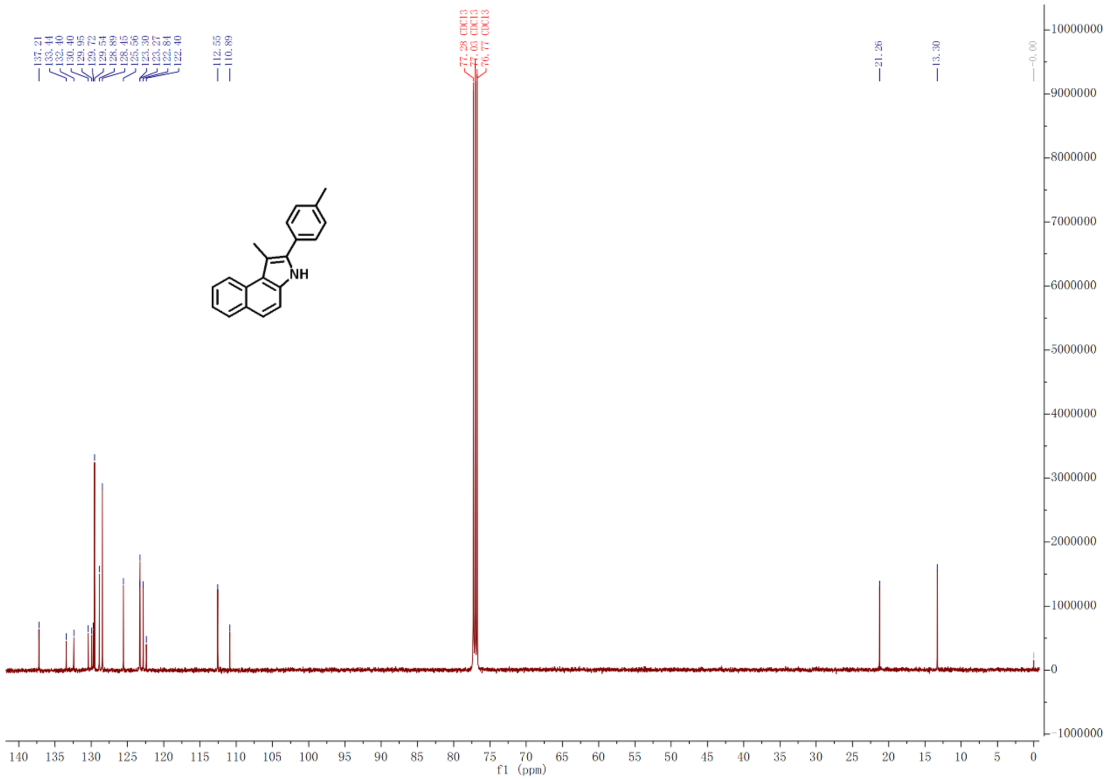

Supplementary Figure. 12 ^{13}C NMR spectrum of Ph-Bd[e] in $CDCl_3$. (126 MHz, Chloroform-*d*)

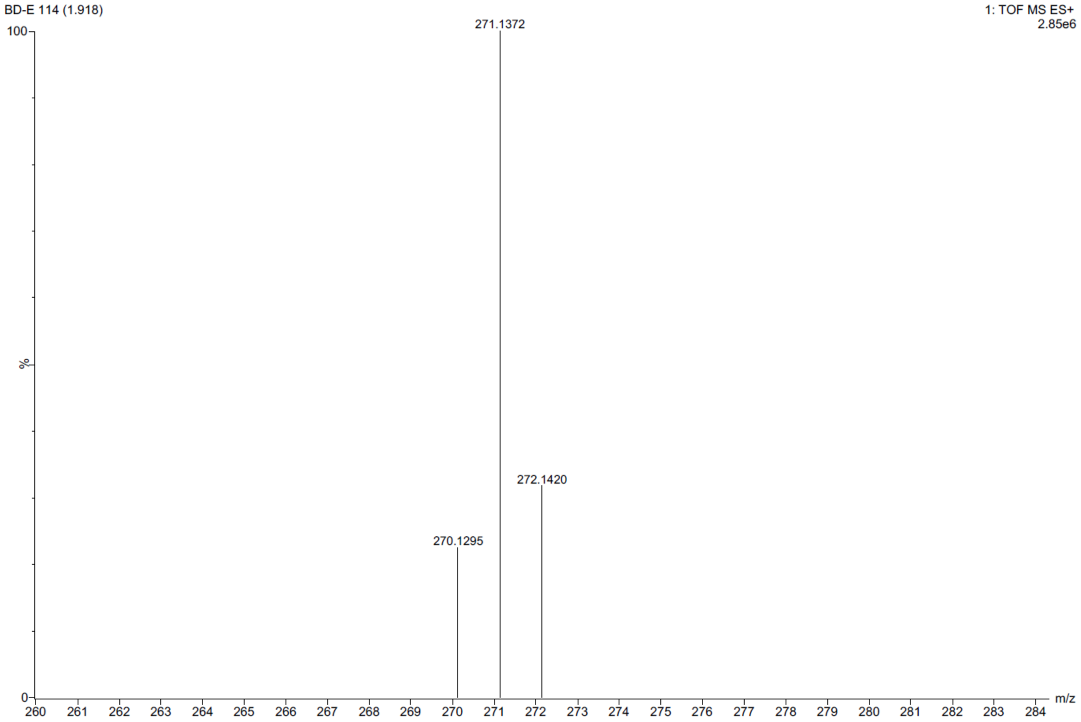

Supplementary Figure. 13 HR-MS spectrum of Ph-Bd[e].

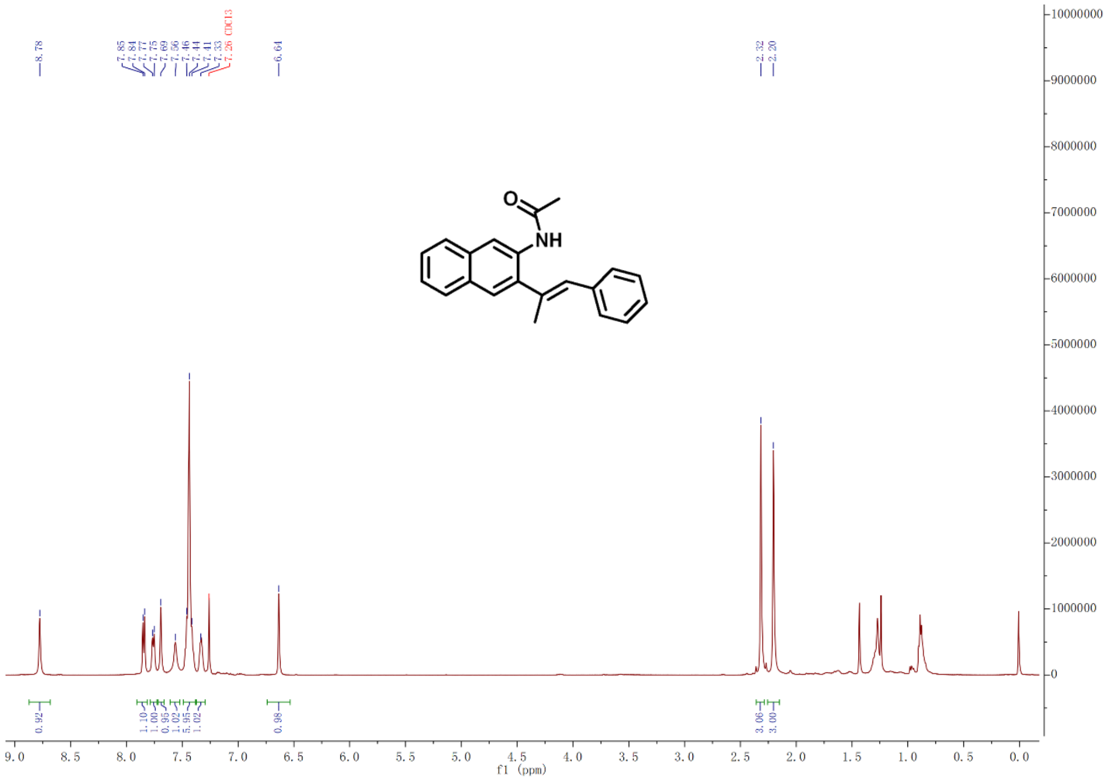

Supplementary Figure. 14 ^{1}H NMR spectrum of 1 in $CDCl_3$. (500 MHz, Chloroform-*d*)

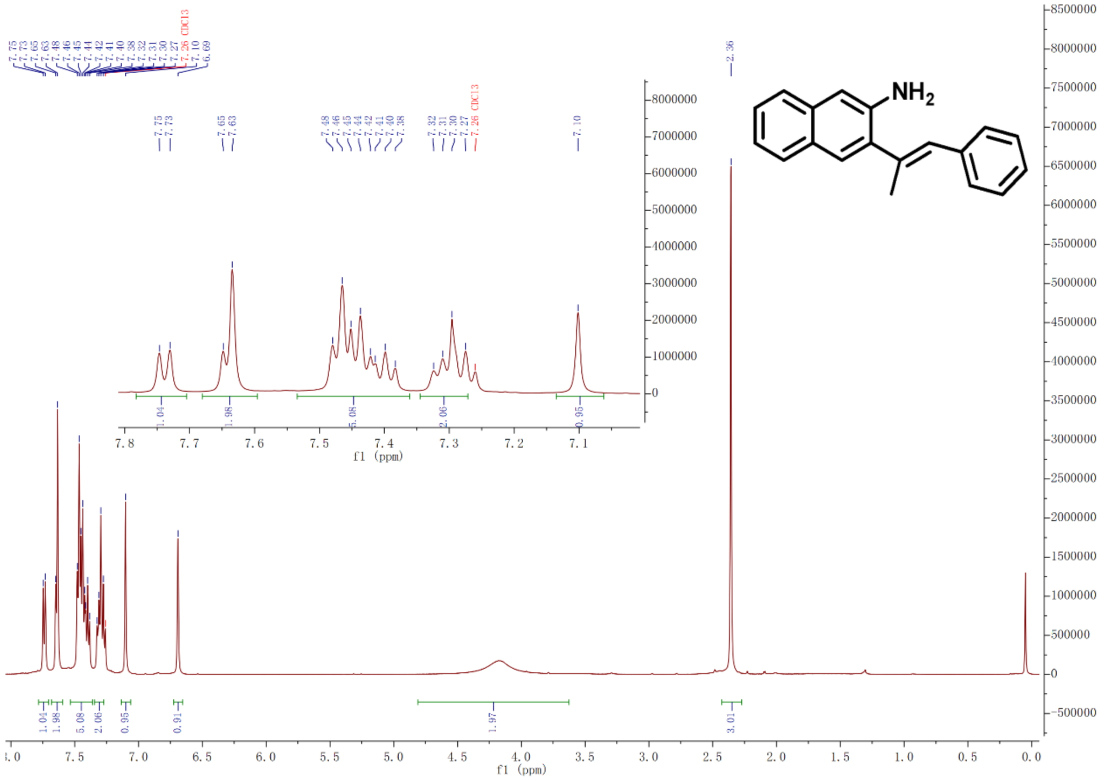

Supplementary Figure. 15 ^{1}H NMR spectrum of 2 in $CDCl_3$. (500 MHz, Chloroform-*d*)

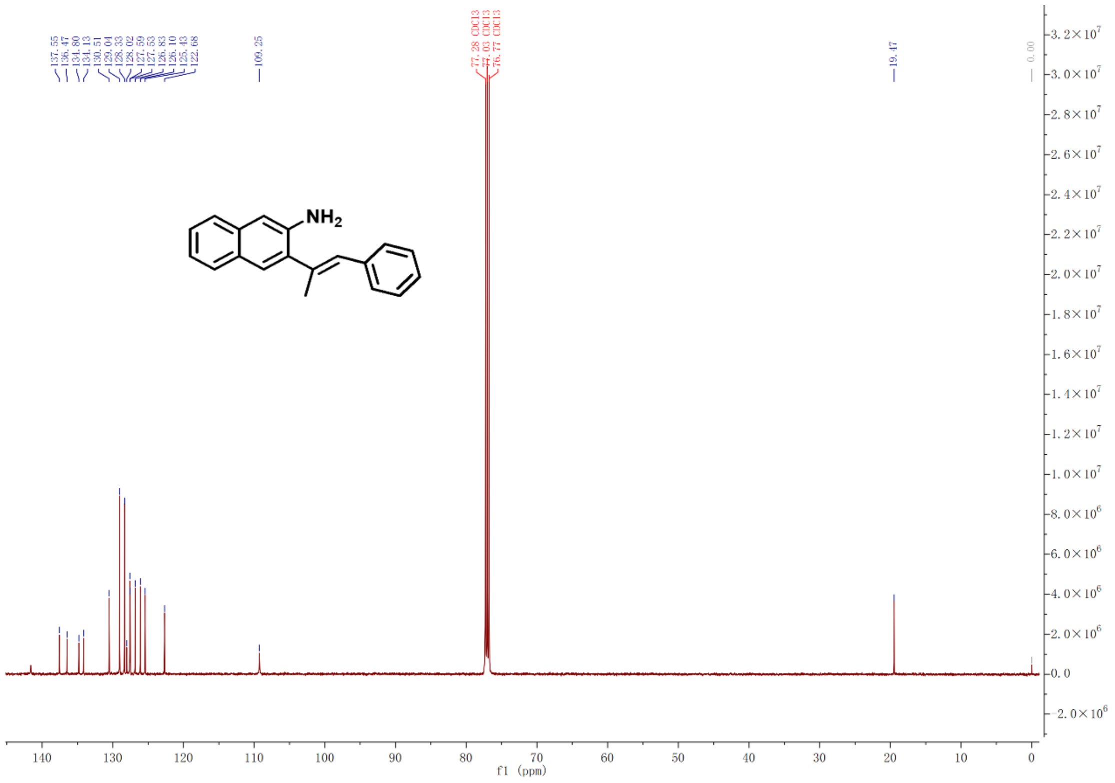

Supplementary Figure. 16 ^{13}C NMR spectrum of 2 in $CDCl_3$. (126 MHz, Chloroform-*d*)

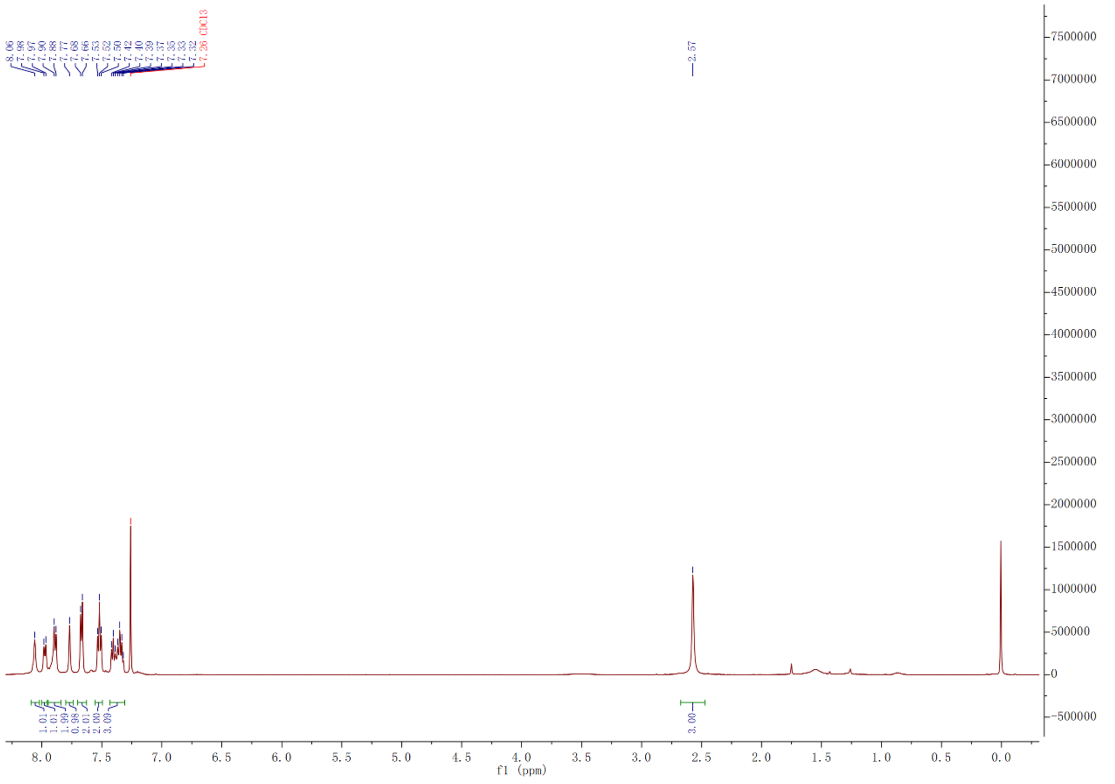

Supplementary Figure. 17 ^{1}H NMR spectrum of Ph-Bd[f] in $CDCl_3$. (500 MHz, Chloroform-*d*)

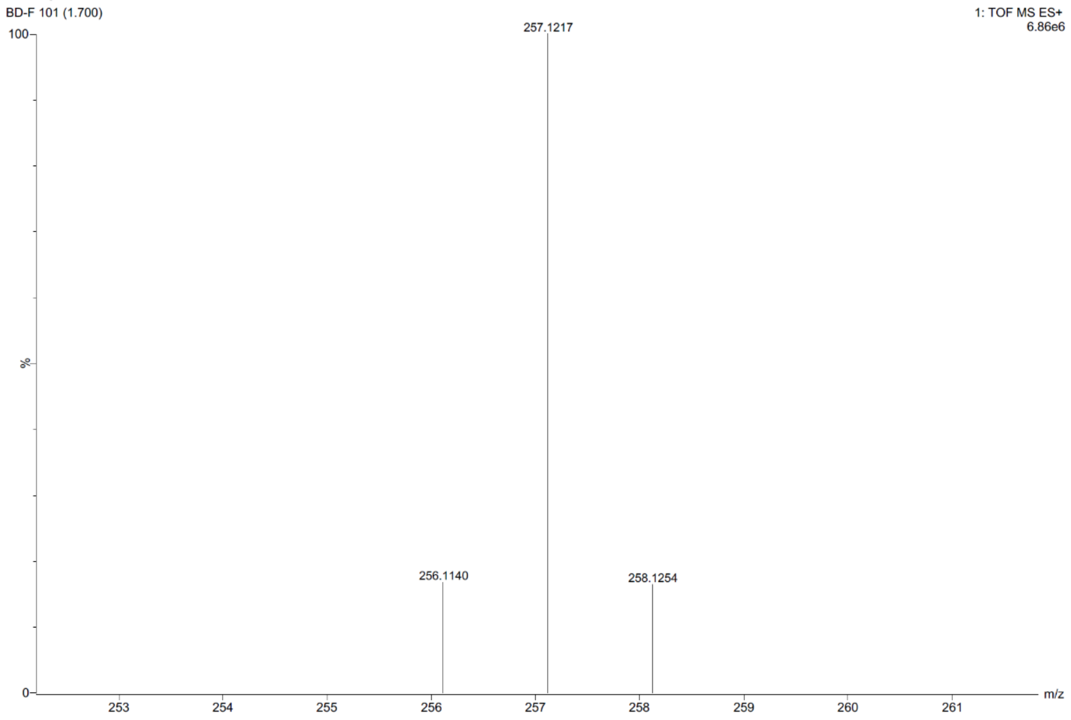

Supplementary Figure. 18 HR-MS spectrum of Ph-Bd[f].

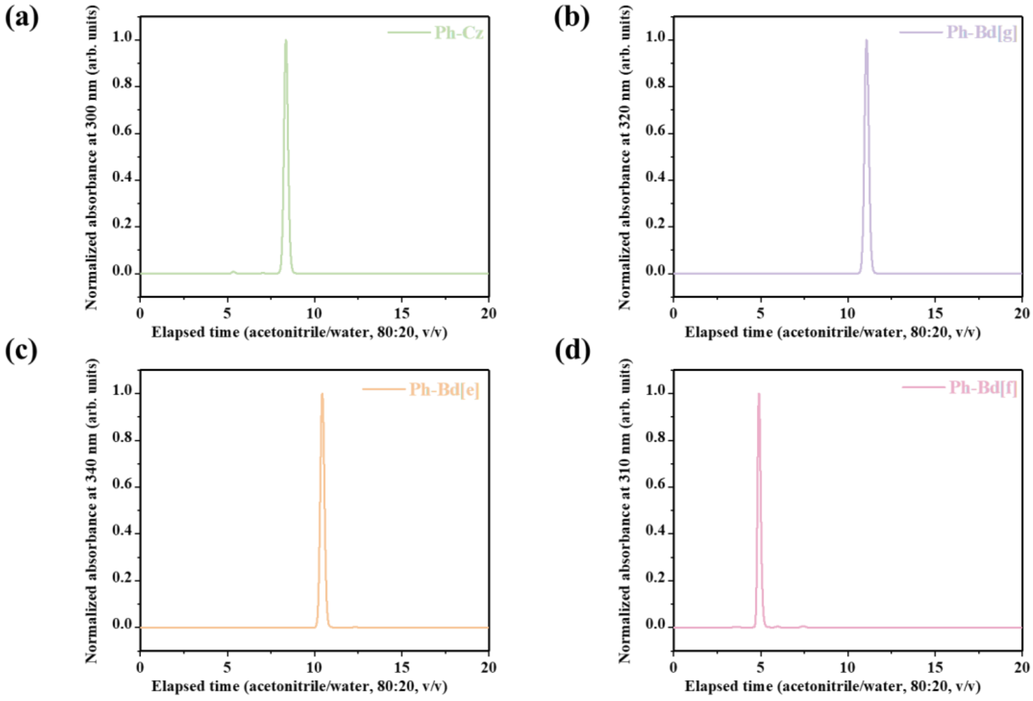

Supplementary Figure. 19 HPLC spectrum of Ph-Cz, Ph-Bd[g], Ph-Bd[e], and Ph-Bd[f].

Supplementary Table 1. Detailed data of the Ph-Cz, Ph-Bd[g], and Ph-Bd[e] single crystal.

Identification code	Ph-Cz	Ph-Bd[g]	Ph-Bd[e]
CCDC Number	2466598	2466760	2360172
Empirical formula	$C_{19}H_{15}N$	$C_{20}H_{17}N$	$C_{20}H_{17}N$
Formula weight	257.32	271.35	271.35
Temperature	273.15 K	273.15 K	273.15 K
Crystal system	orthorhombic	orthorhombic	monoclinic
Space group	$P2_12_12_1$	$P2_12_12_1$	$P2_1/c$
Unit cell dimensions	a = 5.8572(2)Å α= 90° b = 14.5564(5)Å β=90° c = 16.4491(5)Å γ = 90°	a = 5.8797(13)Å α= 90° b = 12.272(3)Å β= 90° c = 20.186(4)Å γ = 90°	a = 7.8544(2)Å α= 90° b = 12.6214(3)Å β=90.7320(10)° c = 29.8691(7)Å γ = 90°
Volume	1402.45(8) $Å^3$	1456.5(6) $Å^3$	2960.79(12)$Å^3$
Z	4	4	8
Density (calculated)	1.219 Mg/m^3	1.273 Mg/m^3	1.217 Mg/m^3
Absorption coefficient	0.539 mm^{-1}	0.544 mm^{-1}	0.536 mm^{-1}
F(000)	544	576	1152
Crystal size	0.12 × 0.1 × 0.08 mm^3	0.12 × 0.1 × 0.08 mm^3	0.22 × 0.2 × 0.18 mm^3
Theta range for data collection	8.11 to 133.186°	8.44 to 133.26°	5.918 to 133.02
Index ranges	-6 ≤ h ≤ 6, -16 ≤ k ≤ 17, -18 ≤ l ≤ 19	-6 ≤ h ≤ 6, -14 ≤ k ≤ 10, -23 ≤ l ≤ 24	-9 ≤ h ≤ 6, -15 ≤ k ≤ 14, -35 ≤ l ≤ 34
Reflections collected	9228	8346	21611
Independent reflections	2430 [R_{int} = 0.0836, R_{sigma} = 0.0653]	2499 [R_{int} = 0.1239, R_{sigma} = 0.0861]	5174 [R_{int} = 0.0348, R_{sigma} = 0.0257]
Final R indices [I>2sigma(I)]	R_1 = 0.0460, wR_2 = 0.1107	R_1 = 0.0741, wR_2 = 0.1809	R_1 = 0.0430, wR_2 = 0.1204
R indices (all data)	R_1 = 0.0516, wR_2 = 0.1138	R_1 = 0.1220, wR_2 = 0.2256	R_1 = 0.0547, wR_2 = 0.1292

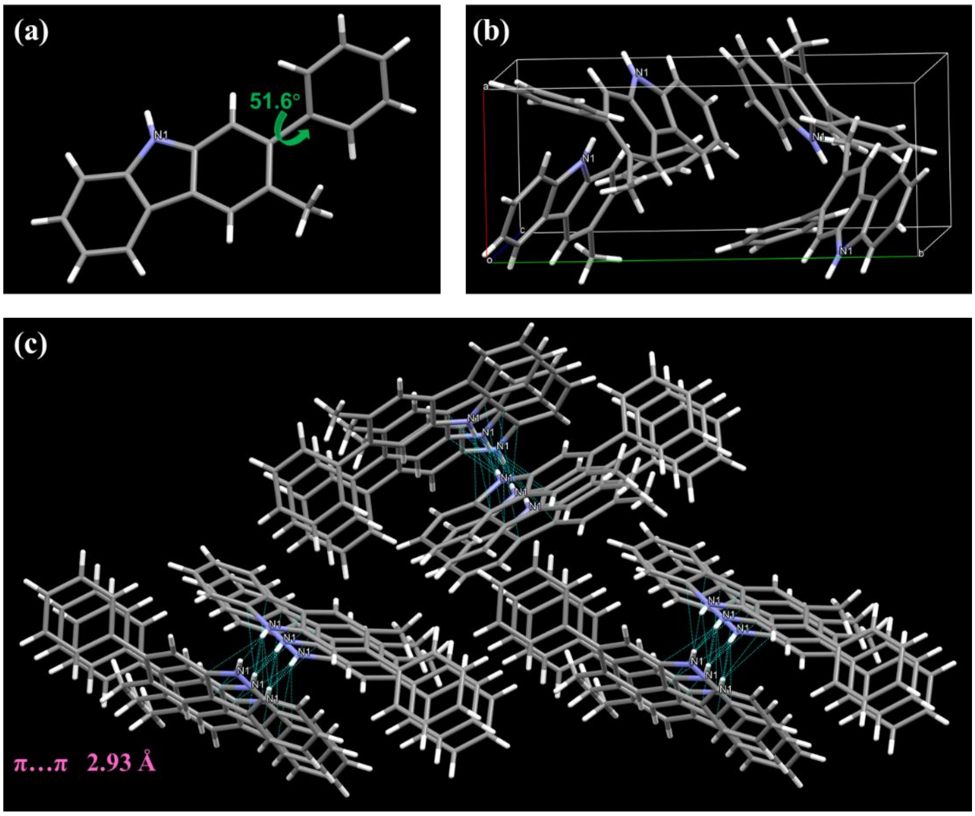

Supplementary Figure. 20 Crystal Structure and Packing of Ph-Cz. (a) Intrinsic molecular geometry of Ph-Cz extracted from single-crystal data. (b) Molecular packing of Ph-Cz within the unit cell. (c) Parallel stacking arrangement of Ph-Cz molecules in the crystal lattice.

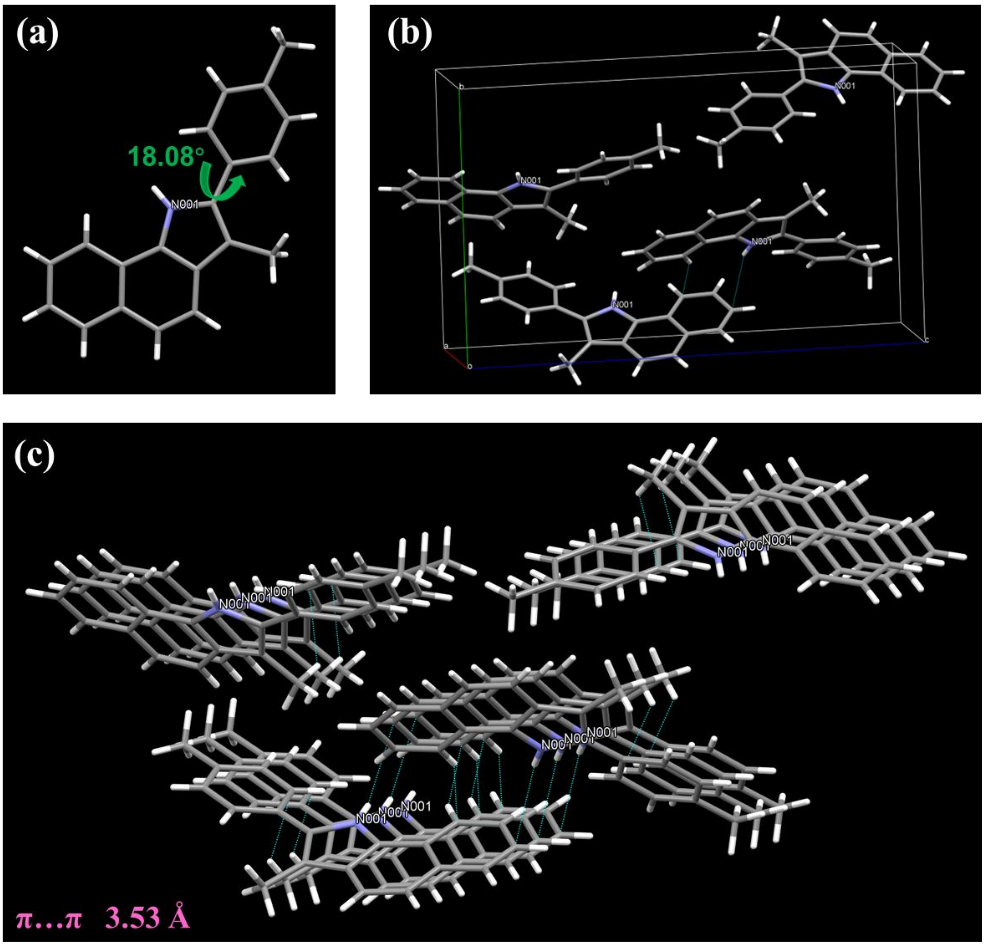

Supplementary Figure. 21 Crystal Structure and Packing of Ph-Bd[g]. (a) Intrinsic molecular geometry of Ph-Bd[g] extracted from single-crystal data. (b) Molecular packing of Ph-Bd[g] within the unit cell. (c) Parallel stacking arrangement of Ph-Bd[g] in the crystal lattice.

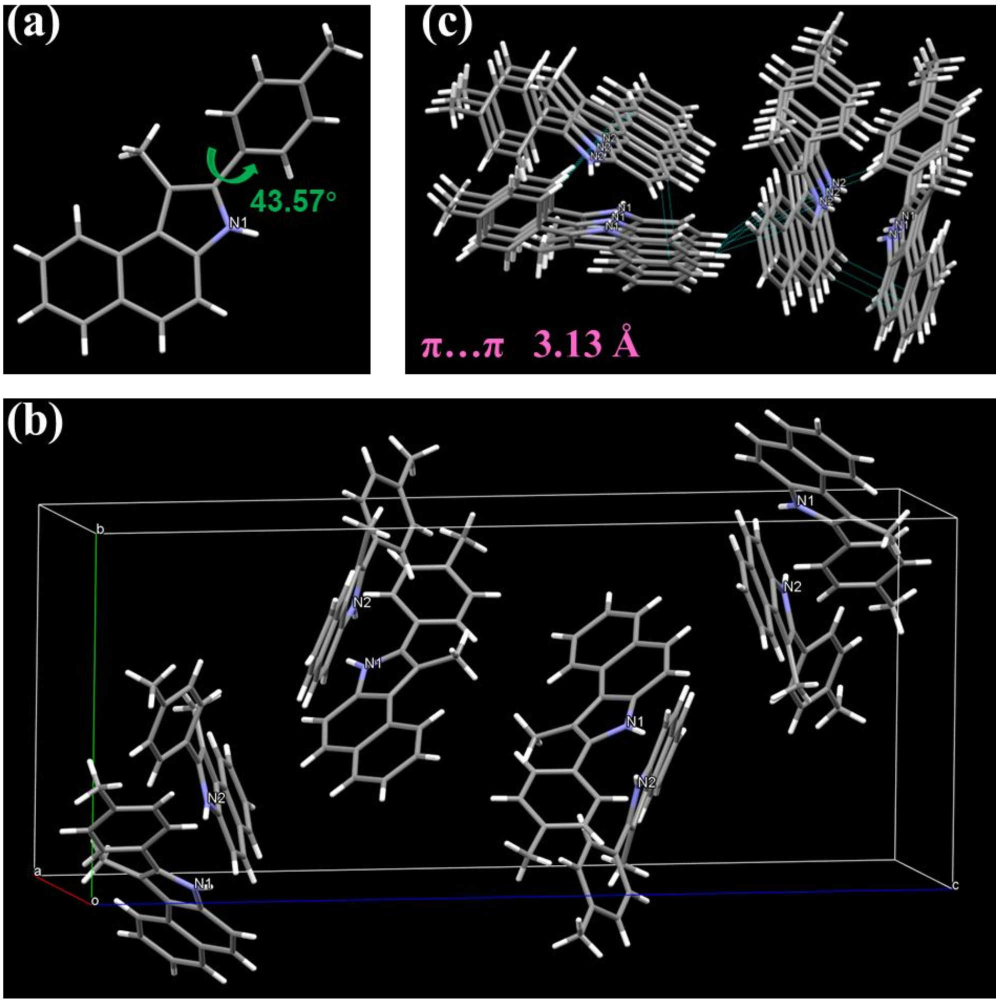

Supplementary Figure. 22 Crystal Structure and Packing of Ph-Bd[e]. (a) Intrinsic molecular geometry of Ph-Bd[e] extracted from single-crystal data. (b) Molecular packing of Ph-Bd[e] within the unit cell. (c) Parallel stacking arrangement of Ph-Bd[e] in the crystal lattice.

Characterisation of material properties

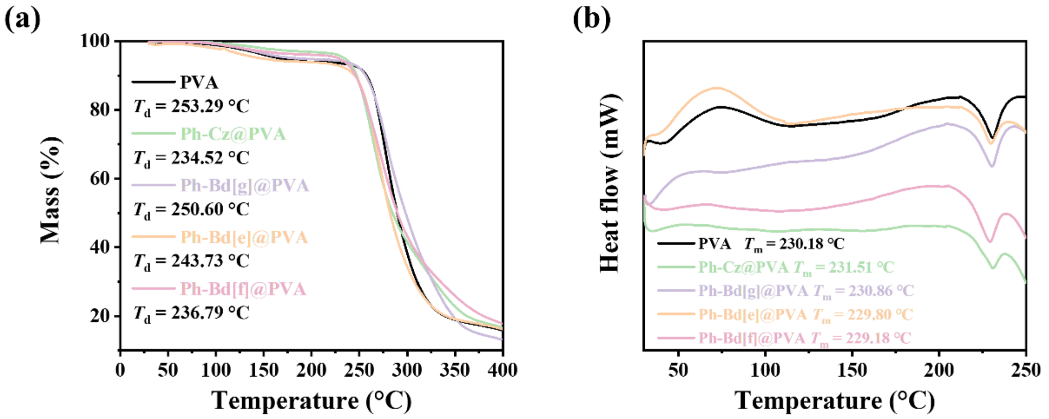

Supplementary Figure. 23 Thermal Properties of Guest-Doped PVA Films. (a) Thermogravimetric analysis (TGA) curves of pure PVA and PVA films doped with four different guest molecules. (b) Differential scanning calorimetry (DSC) curves of pure PVA and the corresponding doped films.

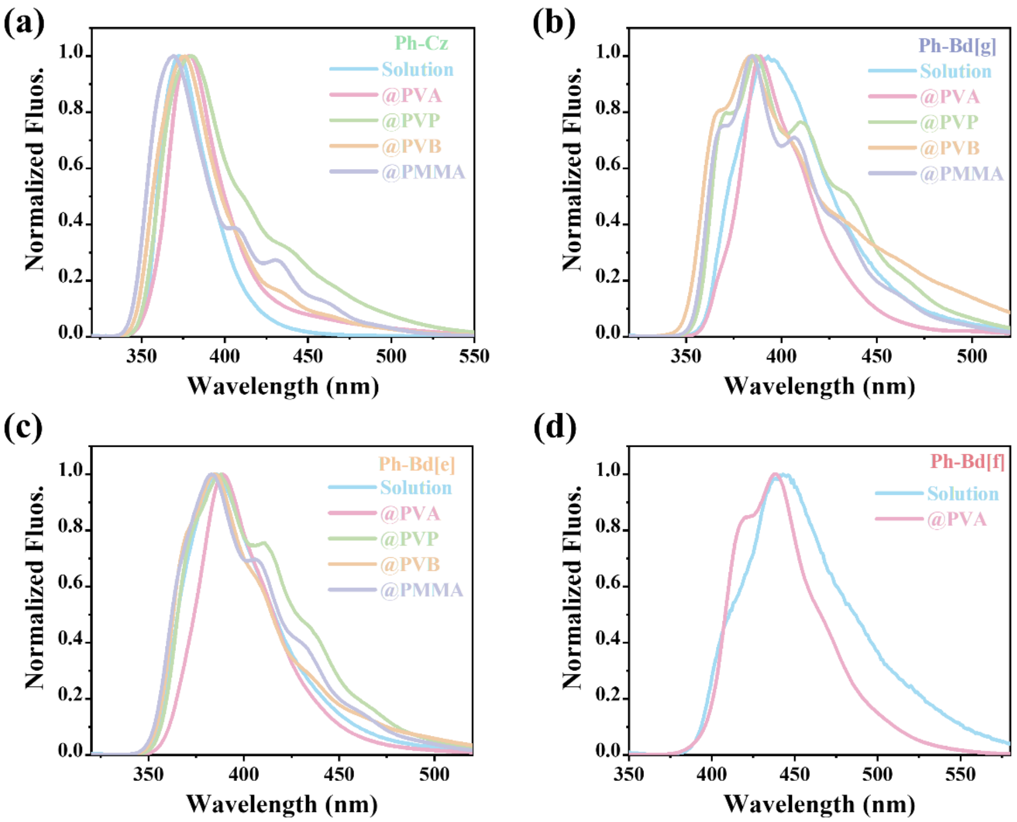

Supplementary Figure. 24 Intrinsic and Matrix-Doped Fluorescence of Guest Molecules. (a) Fluorescence emission spectra of Ph-Cz and its doped films in four different polymer matrices. (b) Fluorescence emission spectra of Ph-Bd[g] and its doped films in four different polymer matrices.

(c) Fluorescence emission spectra of Ph-Bd[e] and its doped films in four different polymer matrices.
(d) Fluorescence emission spectra of Ph-Bd[f] and its doped film in the PVA matrix.

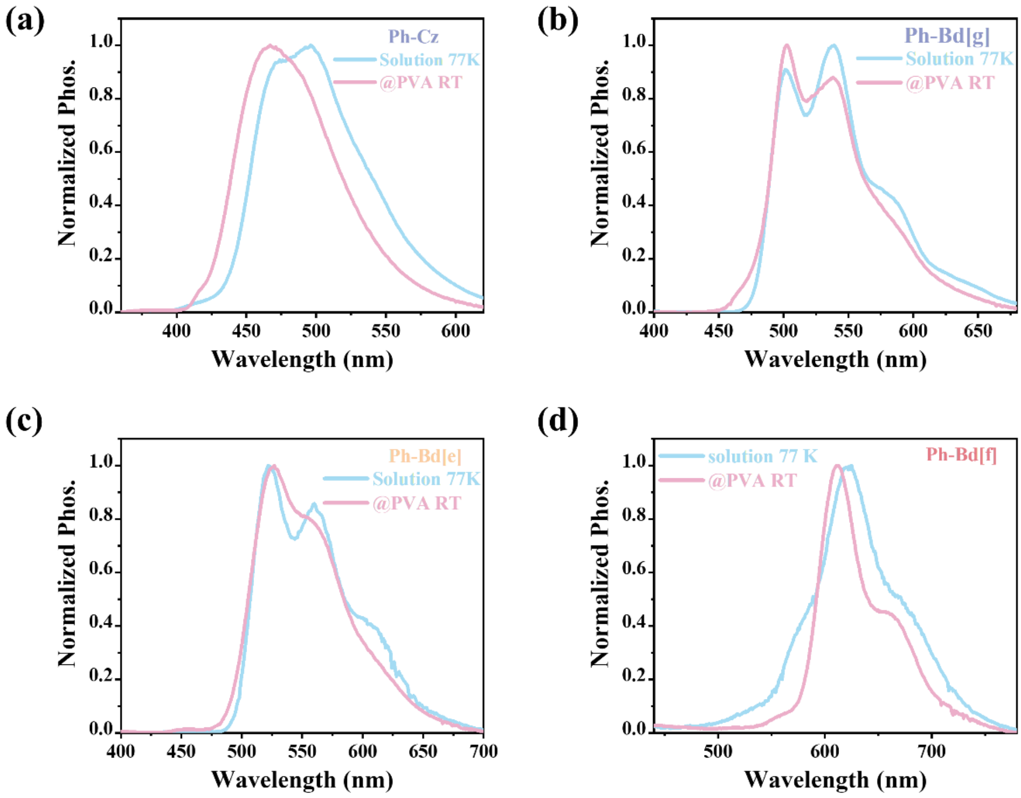

Supplementary Figure. 25 Low-Temperature and PVA-Doped Room-Temperature Phosphorescence. Phosphorescence emission spectra of the four guest molecules at 77 K and their corresponding doped PVA films at room temperature: (a) Ph-Cz, (b) Ph-Bd[g], (c) Ph-Bd[e], and (d) Ph-Bd[f].

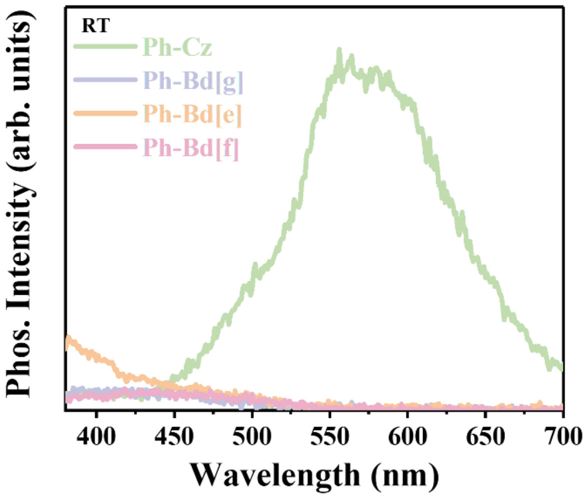

Supplementary Figure. 26 Room-Temperature Phosphorescence of Guest Molecules. Phosphorescence emission spectra of the four guest molecules at room temperature.

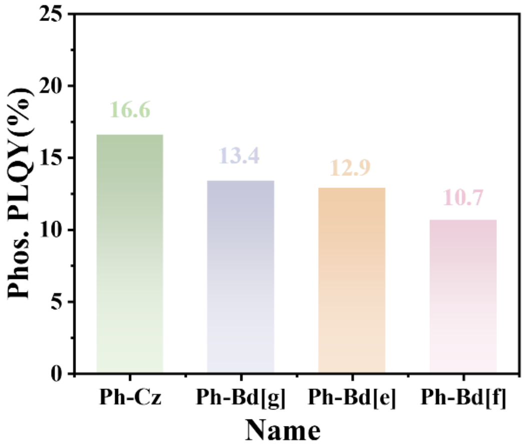

Supplementary Figure. 27 Quantum Yields of Guest-Doped PVA Phosphorescent Films. Phosphorescence quantum yields of PVA films doped with four different guest molecules. (Each bar represents a single measurement.)

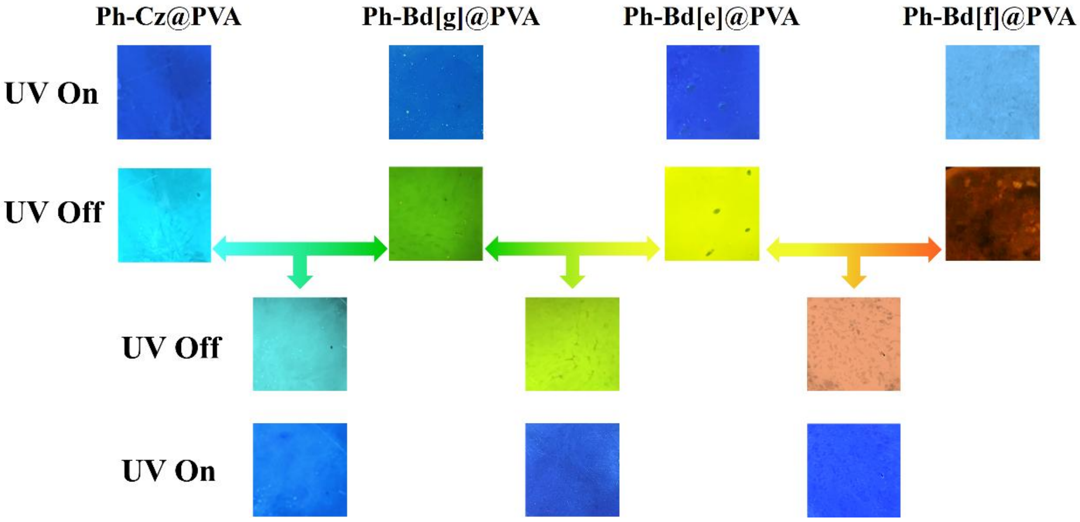

Supplementary Figure. 28 UV On/Off Afterglow of Single and Binary PVA Phosphorescent Films. Photographs of phosphorescent materials under UV on/off conditions. Phosphorescent films prepared by doping four different guest molecules into a PVA matrix (upper panel), and a binary doped PVA composite material that achieves a tunable afterglow color using pairs of adjacent guest molecules (lower panel). Images taken under 365 nm UV irradiation and with the UV turned off.

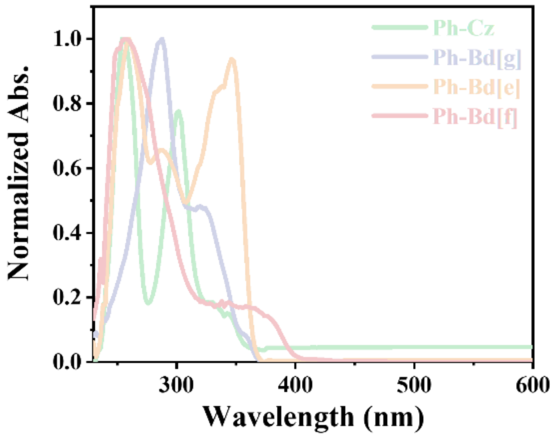

Supplementary Figure. 29 UV-Vis Absorption in THF. UV-vis absorption spectra of the four guest molecules in dilute THF solution.

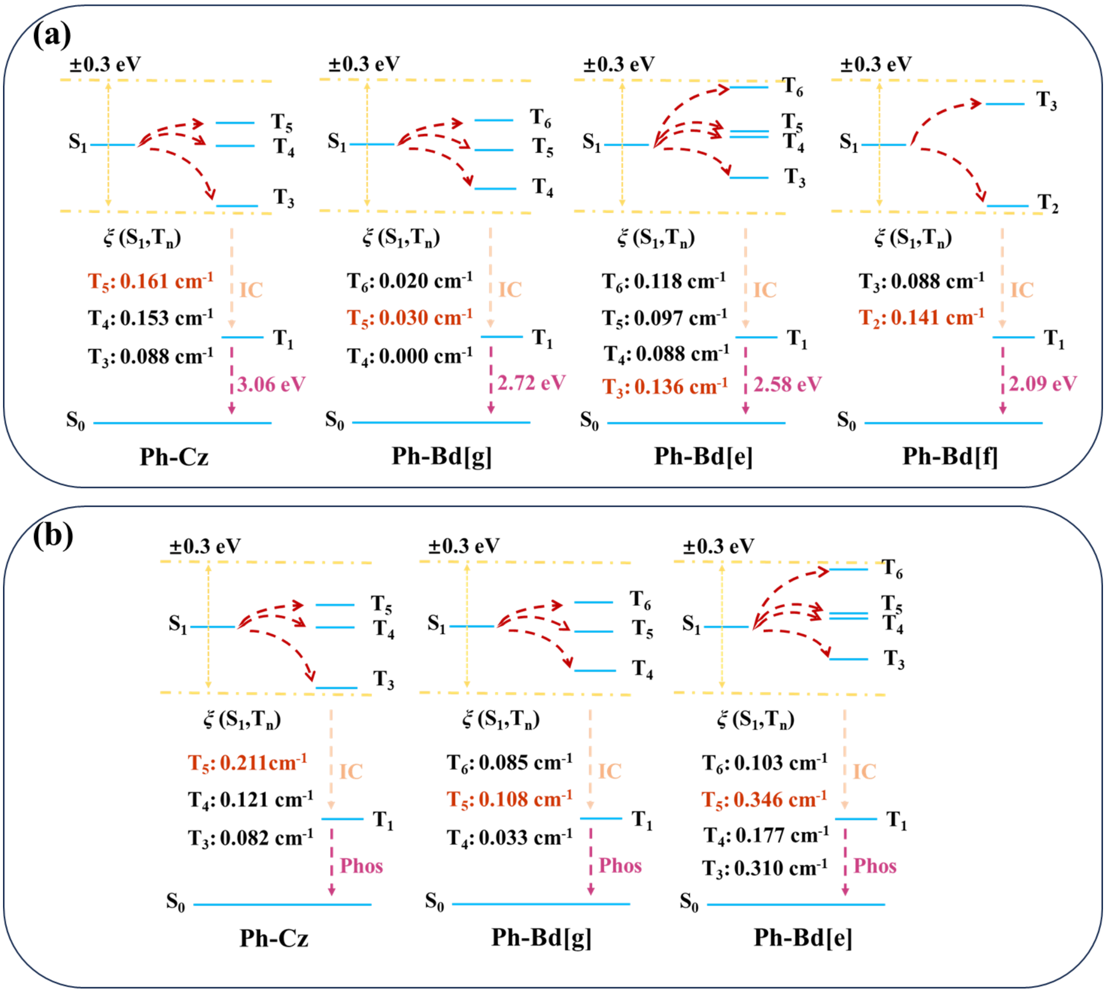

Supplementary Figure. 30 Energy Level Diagrams and SOC-Guided ISC Pathways. (a) Schematic energy level diagrams and corresponding spin-orbit coupling (SOC) constants of the four optimized guest molecules. The red arrows highlight the probable intersystem crossing (ISC)

channels contributing to efficient triplet population. (b) Schematic energy level diagrams and SOC constants of the three guest molecules in their single-crystal states. The red arrows indicate potential ISC pathways facilitating nonradiative transitions between singlet and triplet states.

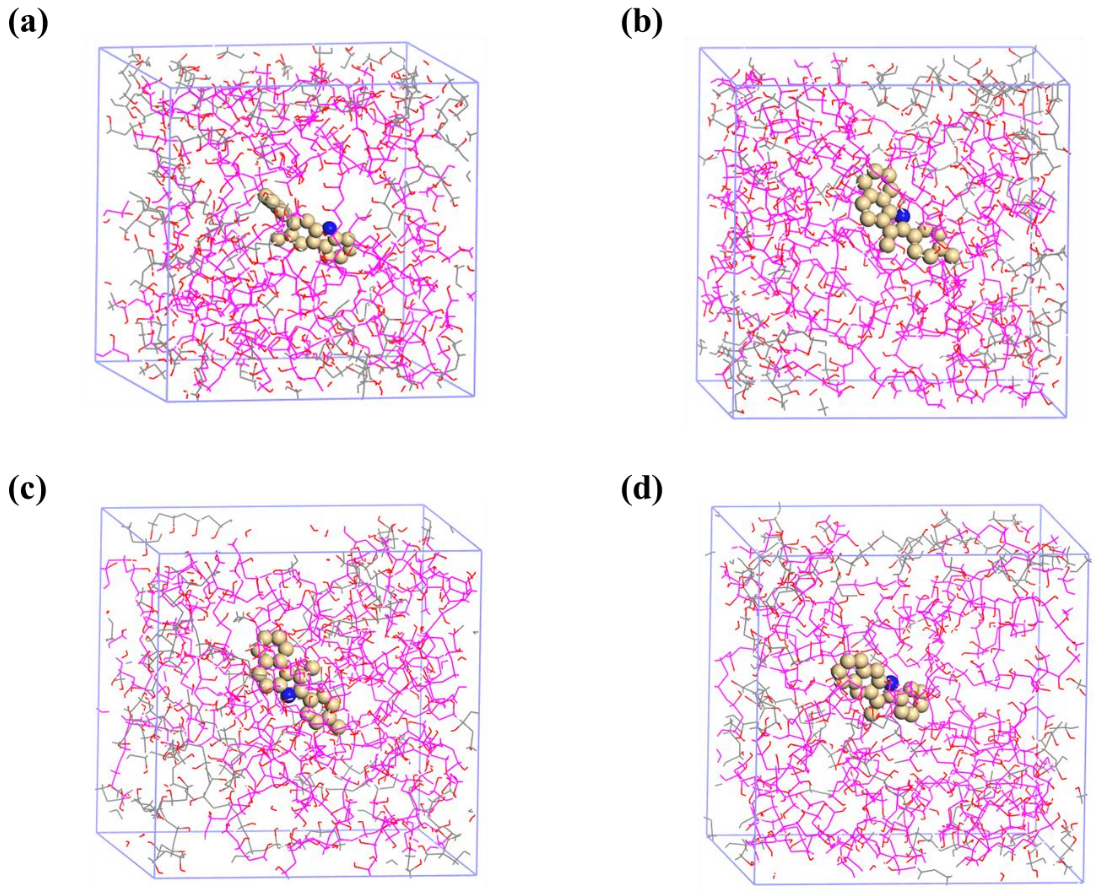

Supplementary Figure. 31 PVA-Embedded Simulation Models of Ph-Cz and Ph-Bd Isomers. The simulation model of Ph-Cz (a), Ph-Bd[g] (b), Ph-Bd[e] (c), Ph-Bd[f] (d), in PVA film.

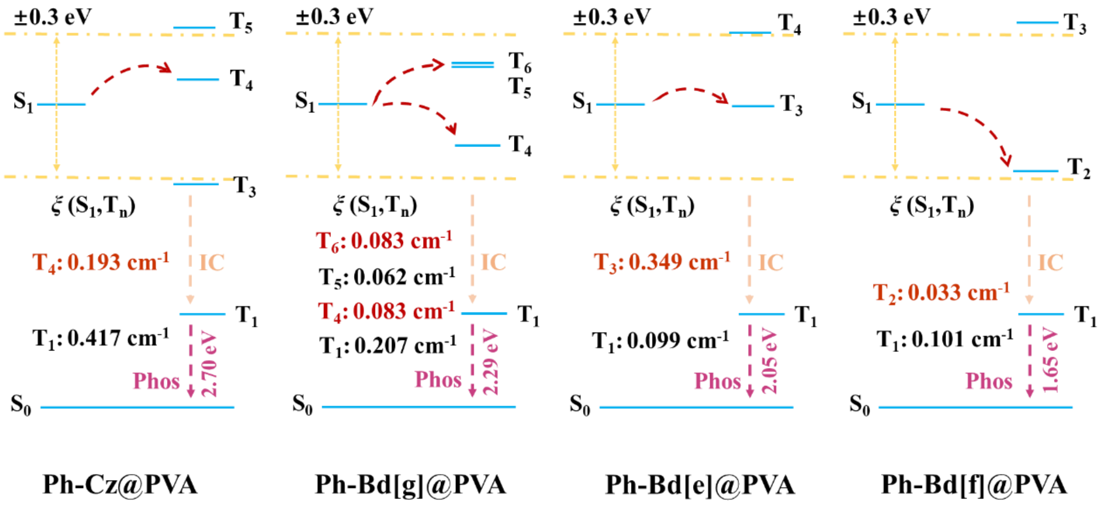

Supplementary Figure. 32 Energy Levels and SOC Analysis of Ph-Cz and Ph-Bd Isomer RTP

Systems. Calculated energy levels and SOC constants of Ph-Cz, Ph-Bd[g], Ph-Bd[e], Ph-Bd[f], RTP systems. Guest molecules were optimized in PVA matrices based on geometries extracted from MD simulations. SOC constants of S_1-T_n transitions available for ISC processes were highlighted in red.

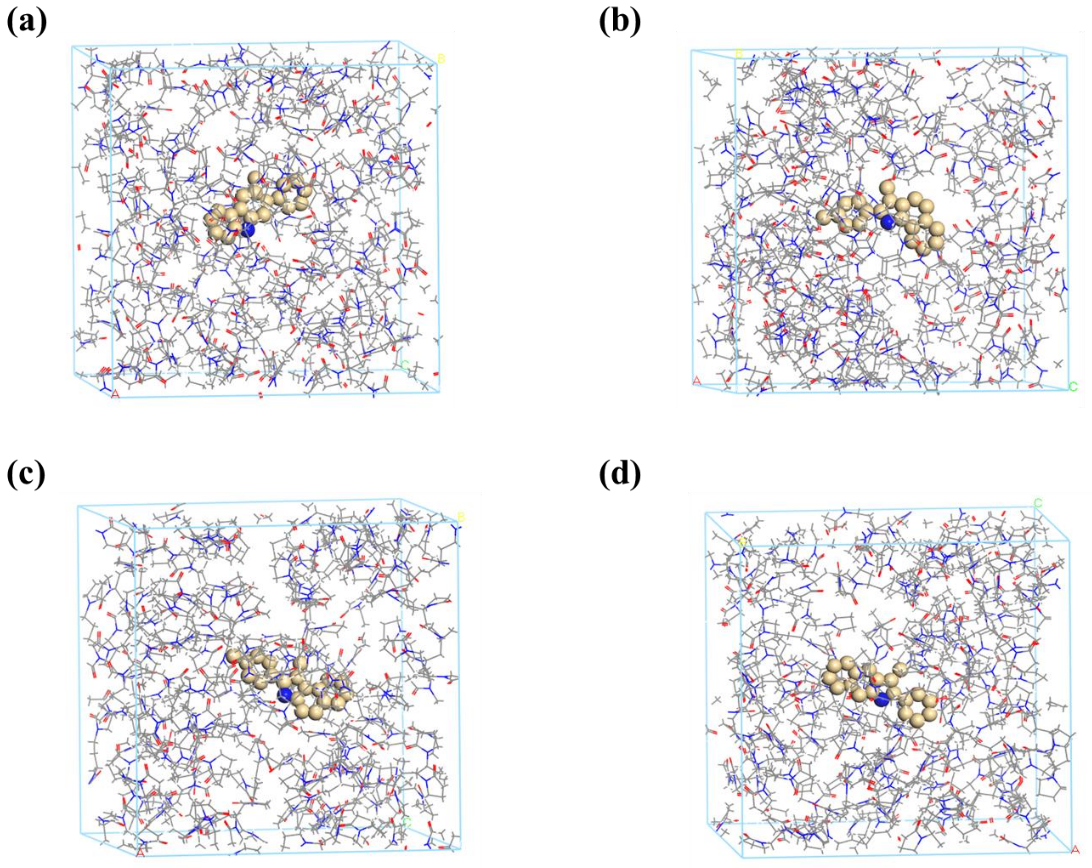

Supplementary Figure. 33 PVP-Embedded Simulation Models of Ph-Cz and Ph-Bd Isomers. The simulation model of Ph-Cz (a), Ph-Bd[g] (b), Ph-Bd[e] (c), Ph-Bd[f] (d), in PVP film.

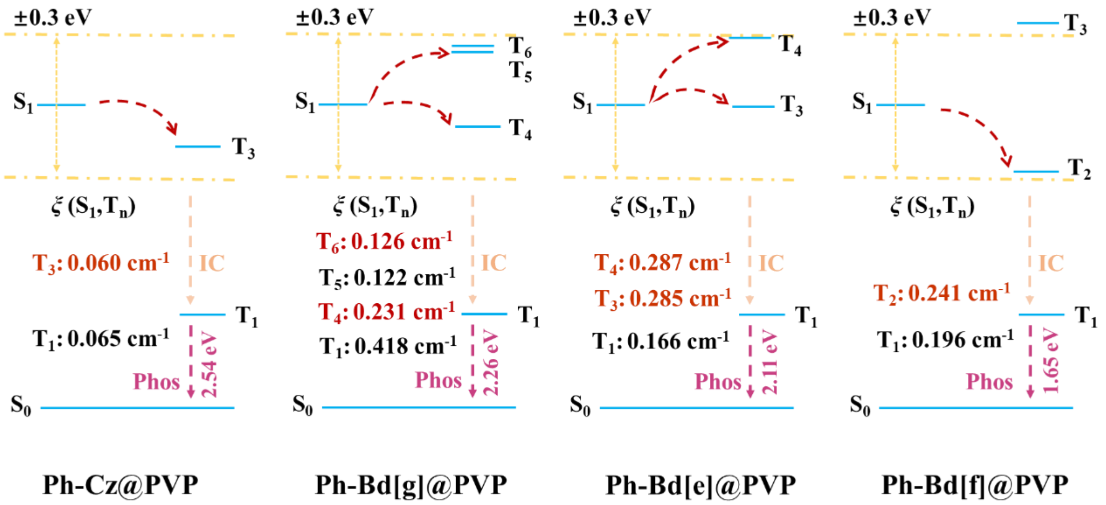

Supplementary Figure. 34 Energy Levels and SOC Analysis of Ph-Cz and Ph-Bd Isomer RTP Systems. Calculated energy levels and SOC constants of Ph-Cz, Ph-Bd[g], Ph-Bd[e], Ph-Bd[f], RTP

systems. Guest molecules were optimized in PVP matrices based on geometries extracted from MD simulations. SOC constants of S_1-T_n transitions available for ISC processes were highlighted in red.

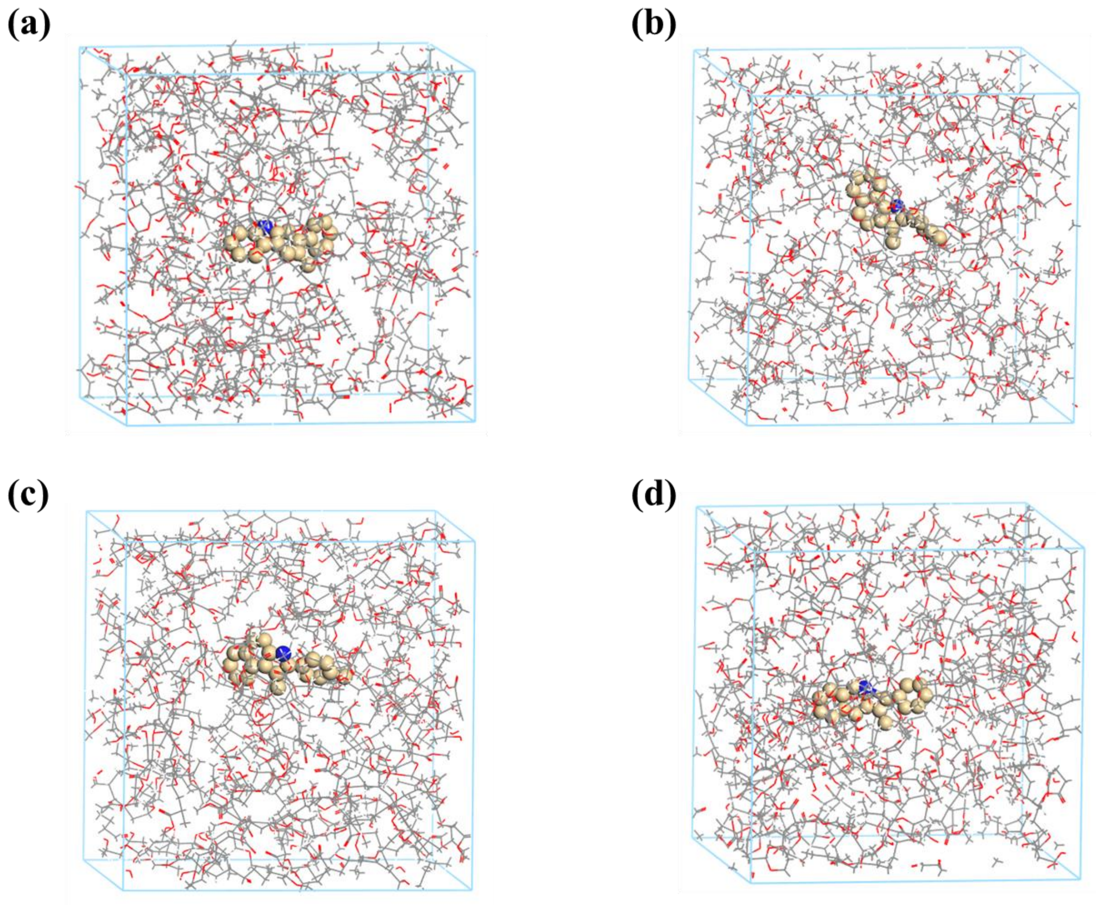

Supplementary Figure. 35 PVB-Embedded Simulation Models of Ph-Cz and Ph-Bd Isomers. The simulation model of Ph-Cz (a), Ph-Bd[g] (b), Ph-Bd[e] (c), Ph-Bd[f] (d), in PVB film.

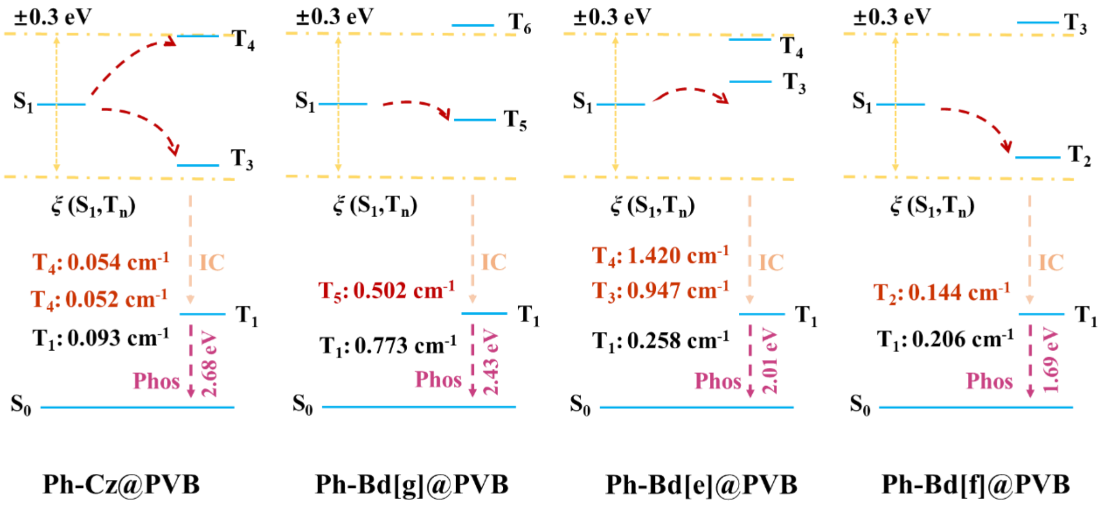

Supplementary Figure. 36 Energy Levels and SOC Analysis of Ph-Cz and Ph-Bd Isomer RTP Systems. Calculated energy levels and SOC constants of Ph-Cz, Ph-Bd[g], Ph-Bd[e], Ph-Bd[f], RTP systems. Guest molecules were optimized in PVB matrices based on geometries extracted from MD simulations. SOC constants of S_1-T_n transitions available for ISC processes were highlighted in red.

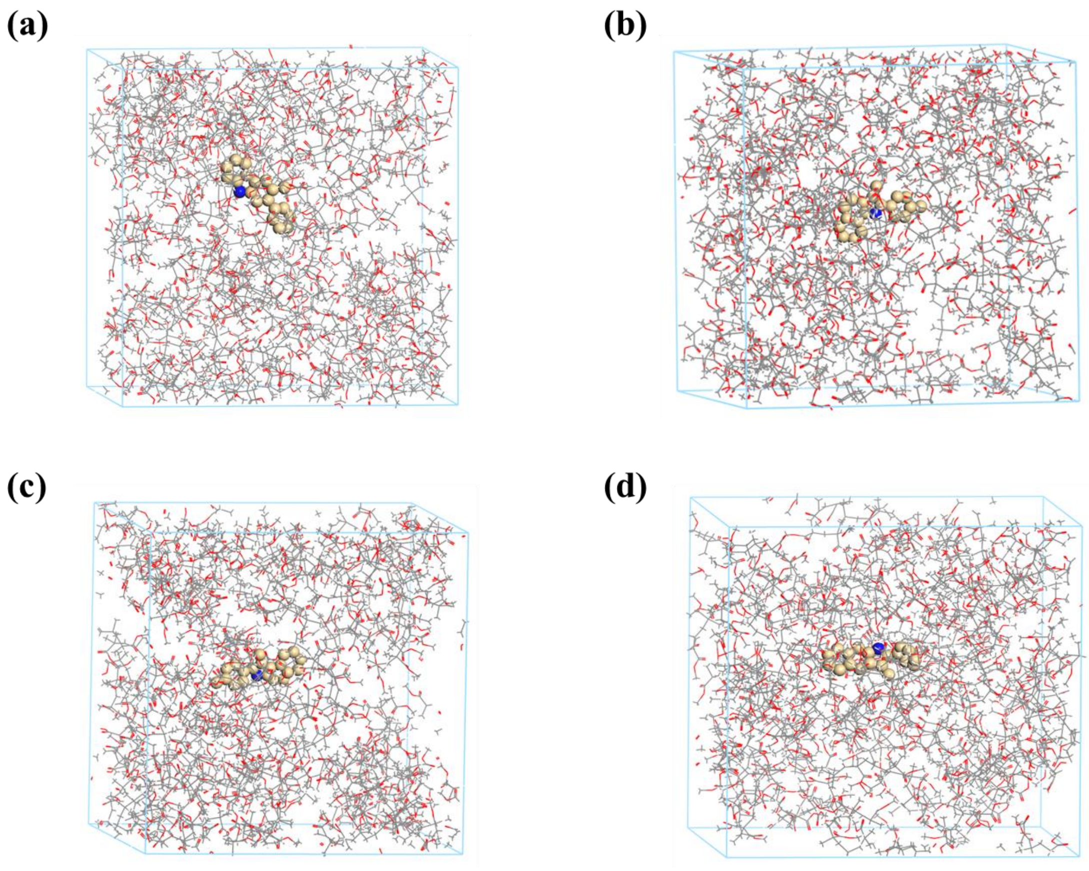

Supplementary Figure. 37 PMMA-Embedded Simulation Models of Ph-Cz and Ph-Bd Isomers. The simulation model of Ph-Cz (a), Ph-Bd[g] (b), Ph-Bd[e] (c), Ph-Bd[f] (d), in PMMA film.

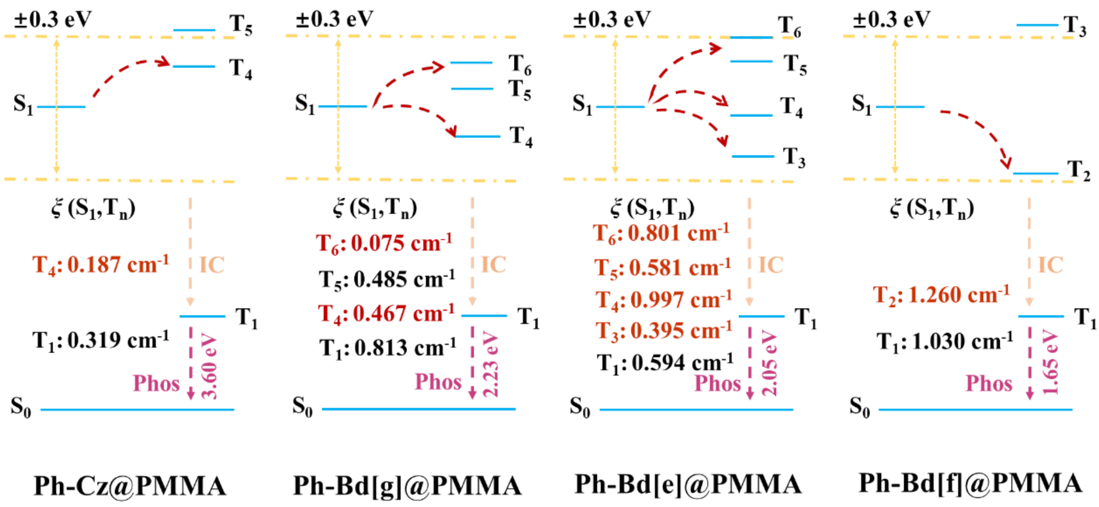

Supplementary Figure. 38 Energy Levels and SOC Analysis of Ph-Cz and Ph-Bd Isomer RTP Systems. Calculated energy levels and SOC constants of Ph-Cz, Ph-Bd[g], Ph-Bd[e], Ph-Bd[f], RTP systems. Guest molecules were optimized in PMMA matrices based on geometries extracted from MD simulations. SOC constants of S_1-T_n transitions available for ISC processes were highlighted in red.

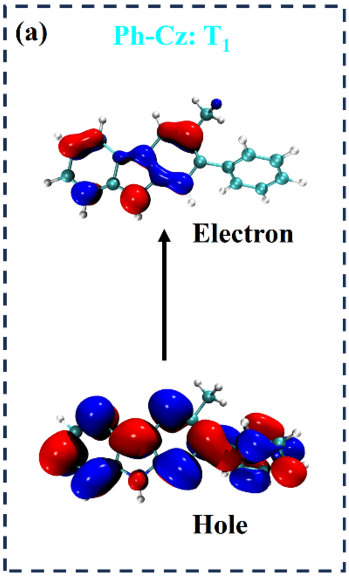

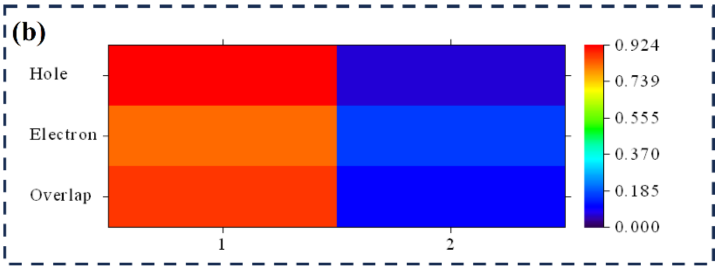

(c)

Contribution of each fragment to hole/electron

Moiety	Hole	Electron	Difference
1(Cz)	92.43%	82.73%	-9.70%
2(Ph,CH_3)	6.80%	16.36%	9.56%

Supplementary Figure. 39 Hole-Electron Composition Analysis of Ph-Cz. Hole/electron composition analysis of Ph-Cz. (a) Hole/electron distribution of T_1 transition. (b) Heat map of hole/electron composition. (c) Contribution of each fragment to hole/electron and charge density difference during transition.

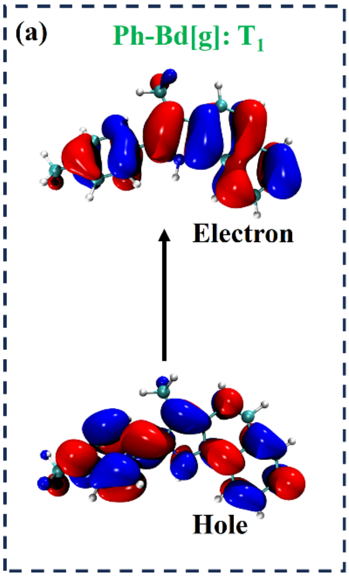

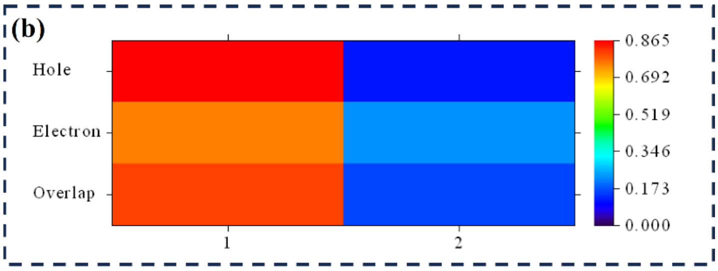

(c)

Contribution of each fragment to hole/electron

Moiety	Hole	Electron	Difference
1(Bd[g])	86.45%	75.75%	-10.71%
2(Ph,CH_3)	11.83%	22.54%	10.71%

Supplementary Figure. 40 Hole-Electron Composition Analysis of Ph-Bd[g]. Hole/electron composition analysis of Ph-Bd[g]. (a) Hole/electron distribution of T_1 transition. (b) Heat map of hole/electron composition. (c) Contribution of each fragment to hole/electron and charge density difference during transition.

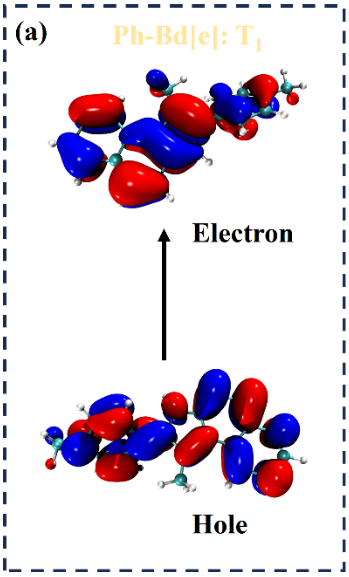

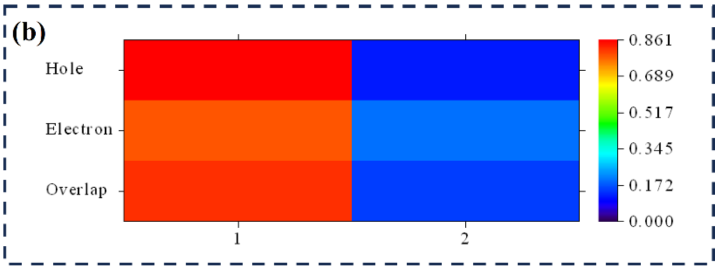

(c)

Contribution of each fragment to hole/electron

Moiety	Hole	Electron	Difference
1(Bd[e])	86.15%	78.74%	-7.40%
2(Ph,CH_3)	12.05%	19.95%	7.90%

Supplementary Figure. 41 Hole-Electron Composition Analysis of Ph-Bd[e]. Hole/electron composition analysis of Ph-Bd[e]. (a) Hole/electron distribution of T_1 transition. (b) Heat map of hole/electron composition. (c) Contribution of each fragment to hole/electron and charge density difference during transition.

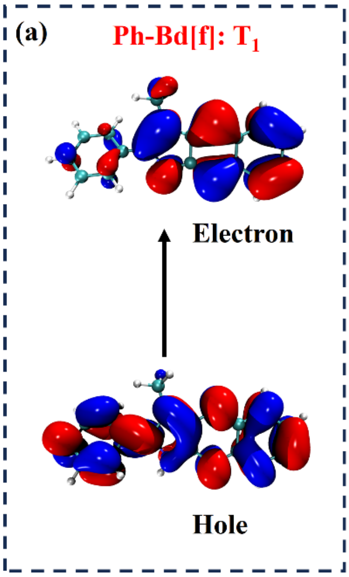

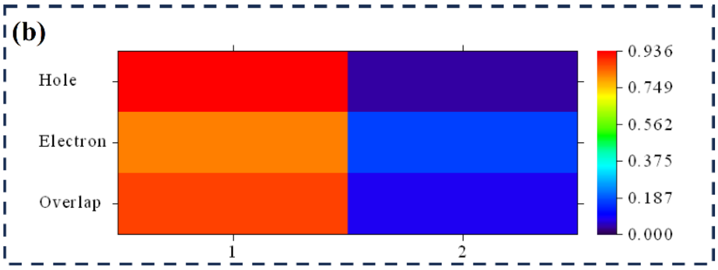

(c) **Contribution of each fragment to hole/electron**

Moiety	Hole	Electron	Difference
1(Bd[f])	93.65%	81.93%	-11.72%
2(Ph,CH_3)	4.14%	17.32%	13.17%

Supplementary Figure. 42 Hole-Electron Composition Analysis of Ph-Bd[f]. Hole/electron composition analysis of Ph-Bd[f]. (a) Hole/electron distribution of T_1 transition. (b) Heat map of hole/electron composition. (c) Contribution of each fragment to hole/electron and charge density difference during transition.

Supplementary Table 2. Single- and three-linear excited state transition configurations of Ph-Cz@PVA obtained by TD-DFT calculations (B3LYP/6-311 d, p). Matched excited states containing the same orbital transition components of S_1 are shown in red.

Excited State	Energy (eV)	Transition configuration (%)
T_1	3.0392	H-1 -> L 73.6%, H -> L+3 6.5%
T_2	3.1971	H -> L 88.9%
T_3	3.6712	H-3 -> L+1 14.9%, H-2 -> L 11.9%, H-1 -> L+1 11.3%, H-2 -> L+2 10.9%, H-3 -> L+2 8.4%, H-1 -> L+2
S_1	3.8451	H -> L 92.9%
T_4	3.8811	H -> L+3 43.0%, H-1 -> L 16.2%, H -> L+1 6.5%, H -> L+2 6.4%, H-2 -> L 6.1%, H-4 -> L 5.2%
T_5	4.0480	H -> L+6 21.5%, H -> L+1 16.3%, H-14 -> L 6.6%, H -> L+2 6.5%, H-4 -> L+3 5.8%, H-1 -> L+9 5.4%

Supplementary Table 3. Single- and three-linear excited state transition configurations of Ph-Bd[g]@PVA obtained by TD-DFT calculations (B3LYP/6-311 d, p). Matched excited states containing the same orbital transition components of S_1 are shown in red.

Excited State	Energy (eV)	Transition configuration (%)

T_1	2.6785	H -> L 59.2%, H-1 -> L+1 24.0%, H -> L+1 6.5%
T_2	3.0220	H -> L+1 30.6%, H-1 -> L+1 26.9%, H -> L 25.5%
T_3	3.4155	H -> L+1 39.7%, H-1 -> L+1 30.4%, H-1 -> L 17.6%
T_4	3.5982	H-1 -> L 70.7%, H -> L+1 14.1%, H-1 -> L+1 7.1%
S_1	3.7614	H -> L+1 66.9%, H-1 -> L 24.2%
T_5	3.7831	H -> L+3 32.1%, H-4 -> L 22.4%, H -> L+2 8.8%, H-7 -> L+2 7.3%
T_6	3.8564	H -> L+2 78.1%, H-4 -> L+2 7.2%

Supplementary Table 4. Single- and three-linear excited state transition configurations of Ph-Bd[e]@PVA obtained by TD-DFT calculations (B3LYP/6-311 d, p). Matched excited states containing the same orbital transition components of S_1 are shown in red.

Excited State	Energy (eV)	Transition configuration (%)
T_1	2.5842	H -> L 84.6%
T_2	3.2348	H -> L+1 56.7%, H-2 -> L 20.1%, H-3 -> L+2 5.8%
T_3	3.5887	H-1 -> L 83.7%
S_1	3.7607	H -> L 95.7%
T_4	3.8235	H -> L+3 22.6%, H-3 -> L+2 13.2%, H-2 -> L+1 13.1%, H-4 -> L 9.5%, H-1 -> L+3 9.4%, H-1 -> L+1 5.6%, H-1 -> L 5.1%
T_5	3.9479	H -> L+2 78.6%, H-2 -> L+2 8.4%
T_6	4.0104	H-1 -> L+1 25.5%, H -> L+3 22.9%, H -> L+1 17.5%, H-1 -> L+3 8.7%, H-2 -> L 6.8%

Supplementary Table 5. Single- and three-linear excited state transition configurations of Ph-Bd[f]@PVA obtained by TD-DFT calculations (B3LYP/6-311 d, p). Matched excited states containing the same orbital transition components of S_1 are shown in red.

Excited State	Energy (eV)	Transition configuration (%)
T_1	2.0462	H -> L 90.5%
T_2	2.9729	H-1 -> L 56.7%, H -> L+2 24.3%, H-2 -> L 7.4%
S_1	3.1972	H -> L 97.4%
T_3	3.4357	H-1 -> L 34.9%, H -> L+2 31.7%, H-2 -> L 19.9%

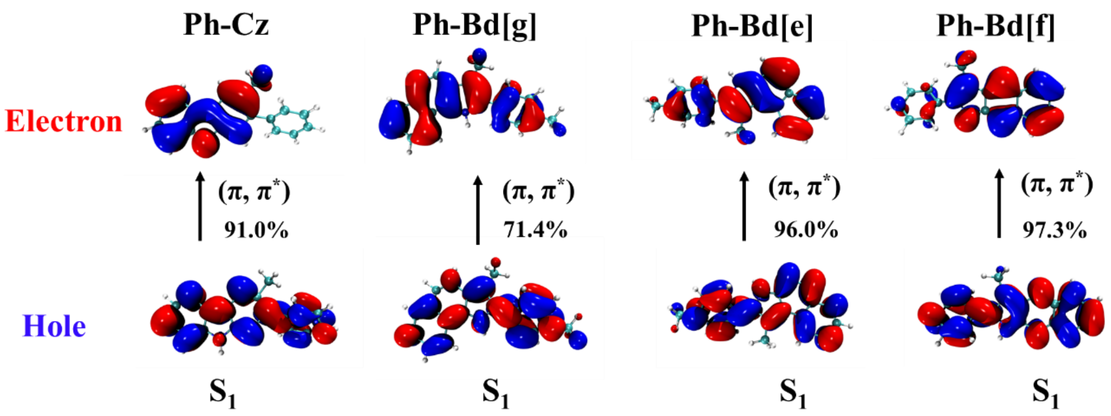

Supplementary Figure. 43 Hole-Electron Distribution in S_1 Transitions of RTP Systems. Analysis of representative hole/electron distributions in the S_1 transitions of existing room-temperature phosphorescence (RTP) systems. "Hole" denotes the hole distribution; " Electron" denotes the electron distribution.

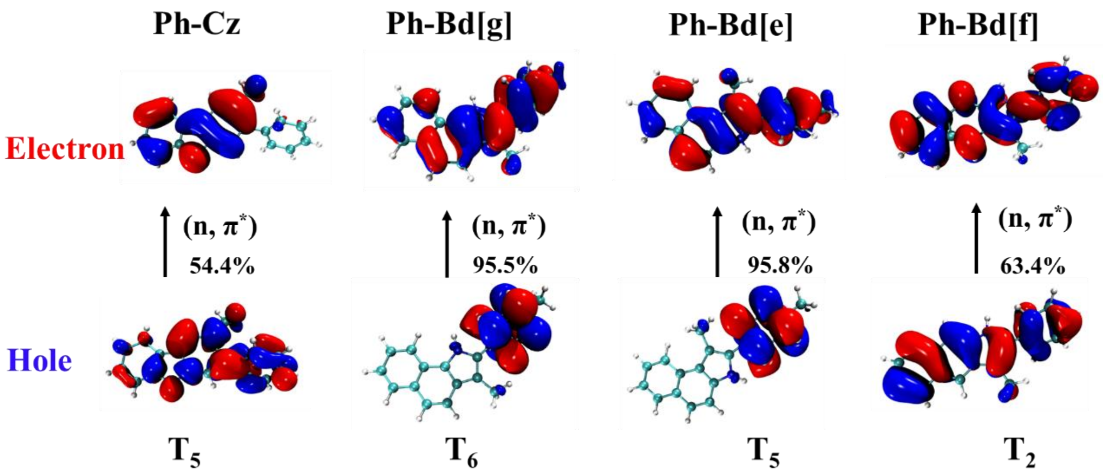

Supplementary Figure. 44 Hole-Electron Distribution in T_n Transitions of Representative RTP Systems. Analysis of representative hole/electron distributions in the T_n transitions of existing room-temperature phosphorescence (RTP) systems. "Hole" denotes the hole distribution; " Electron" denotes the electron distribution.

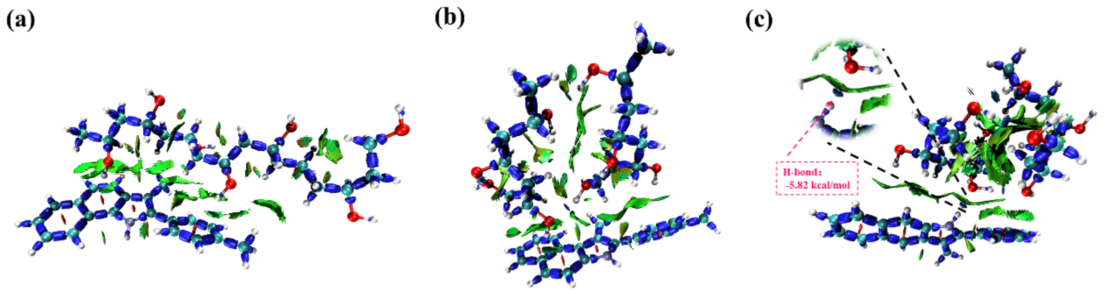

Supplementary Figure. 45 IRI Analysis of Noncovalent Interactions between Ph-Bd Isomers and PVA. Interaction region indicator (IRI) analysis revealing non-covalent interactions between (a) Ph-Bd[g], (b) Ph-Bd[e], (c) Ph-Bd[f], and the PVA matrix, including hydrogen bonding and van der Waals forces.

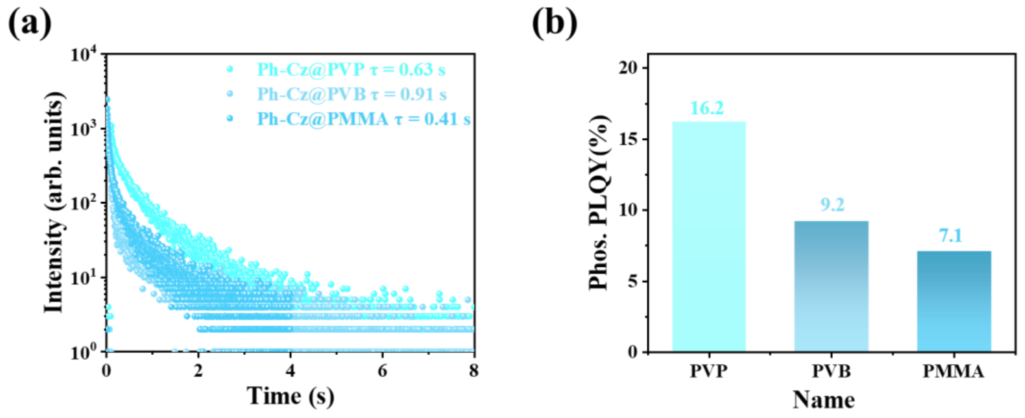

Supplementary Figure. 46 Phosphorescence of Ph-Cz in Different Matrices. (a) Phosphorescence lifetimes of Ph-Cz doped in different polymer matrices. (b) Phosphorescence quantum yields of Ph-Cz doped in different polymer matrices. (Each bar represents a single measurement.)

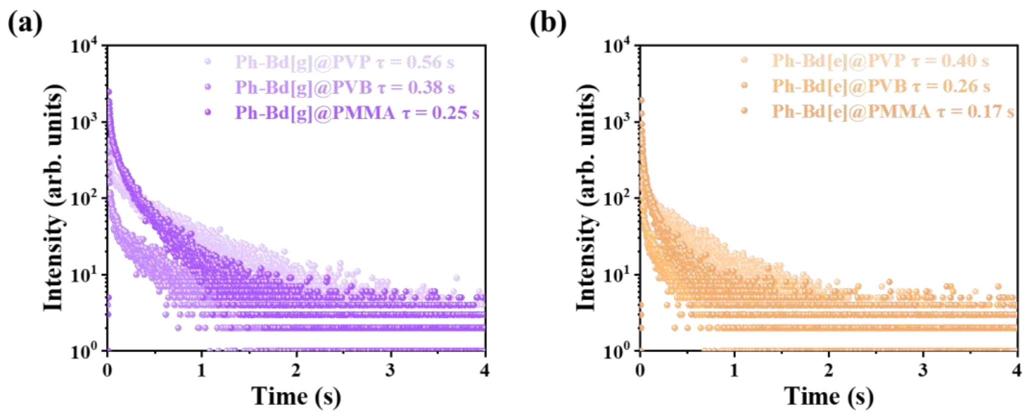

Supplementary Figure. 47 Polymer-Dependent Phosphorescence Lifetimes of Ph-Bd Isomers. Phosphorescence lifetimes of materials prepared by (a) Ph-Bd[g], (a) Ph-Bd[e], doped with different polymeric substrates.

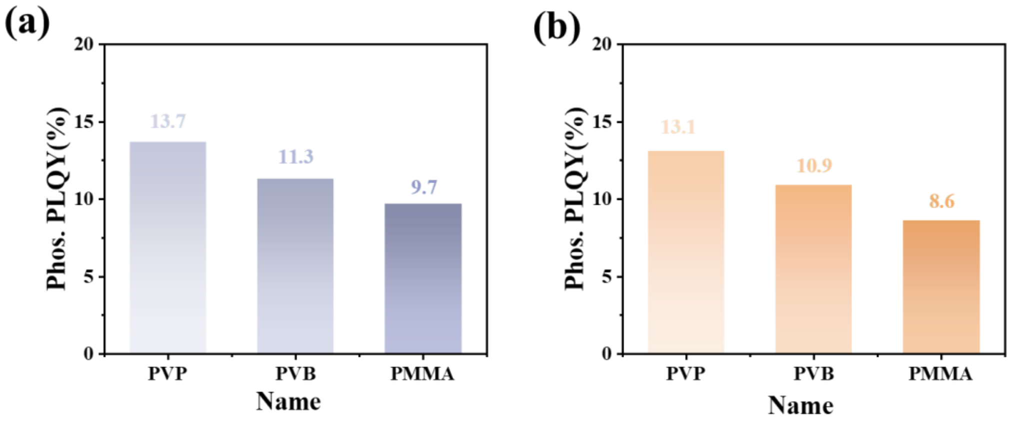

Supplementary Figure. 48 Polymer-Dependent Phosphorescence Quantum Yields of Ph-Bd Isomers. Phosphorescence quantum yields of materials prepared by (a) Ph-Bd[g], (a) Ph-Bd[e], doping with different polymeric substrates. (Each bar represents a single measurement.)

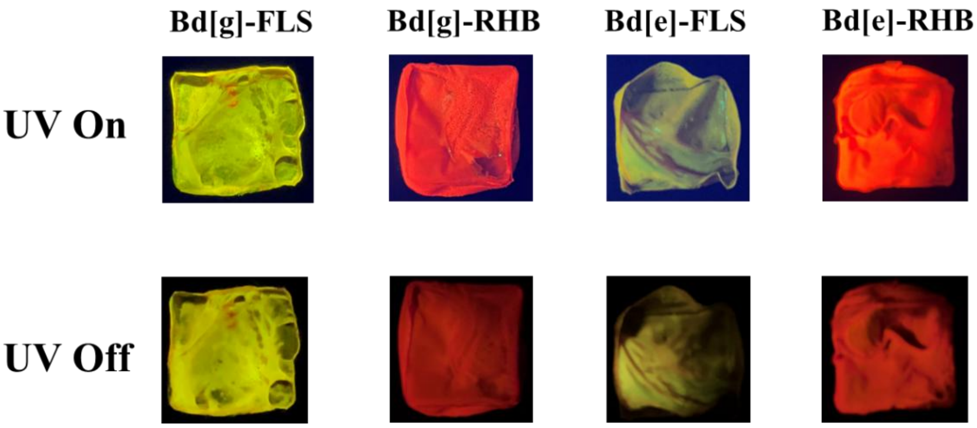

Supplementary Figure. 49 UV-Responsive Afterglow of Bd Isomer Systems in PVA. UV On and UV Off photographs of Bd[g]-FLS, Bd[g]-RHB, Bd[e]-FLS and Bd[e]-RHB under 365 nm torch excitation in PVA substrates.

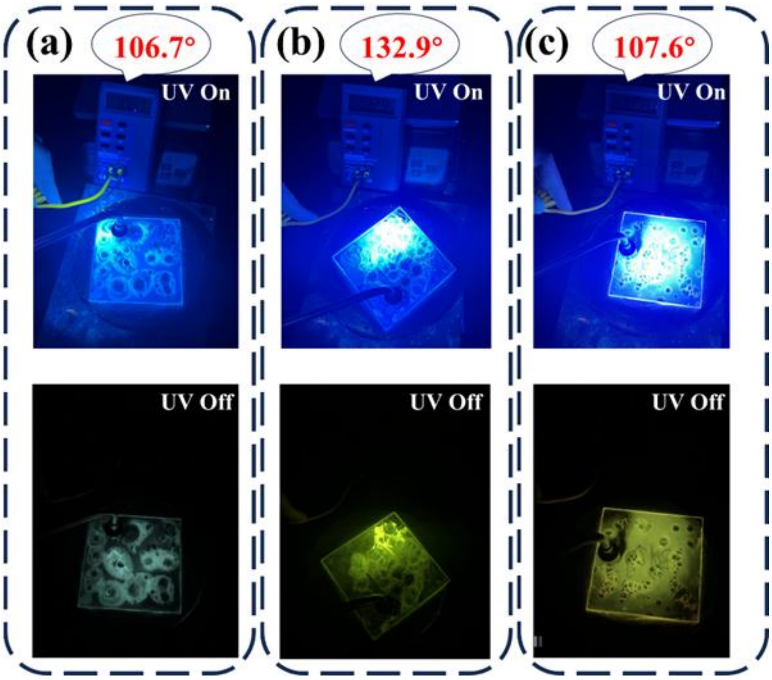

Supplementary Figure. 50 Heat-Resistant Phosphorescence of PVP-Doped Films. Photographs of Ph-Cz@PVP (a), Ph-Bd[g]@PVP (b), and Ph-Bd[e]@PVP (c) samples phosphorescence heat-resistant, taken under UV light on and UV light off conditions, respectively.

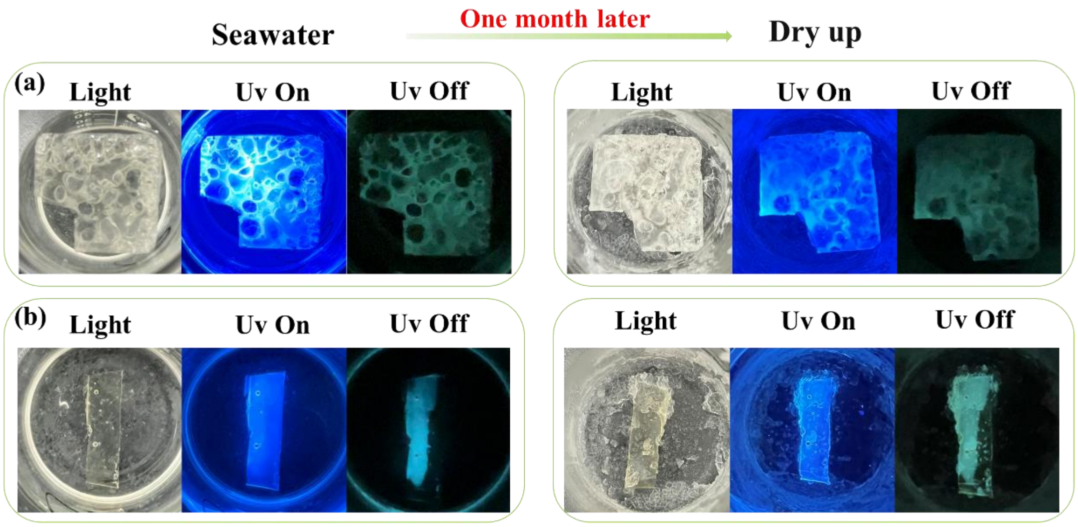

Supplementary Figure. 51 Long-Term Seawater Exposure and Afterglow Retention of Ph-Cz Films. Photographs of Ph-Cz@PMMA (a) and Ph-Cz@PVB (b) samples immediately after immersion in seawater and after one month of seawater exposure followed by evaporation-induced solid residue formation, captured under daylight, UV excitation (UV on), and after UV excitation is turned off (UV off).

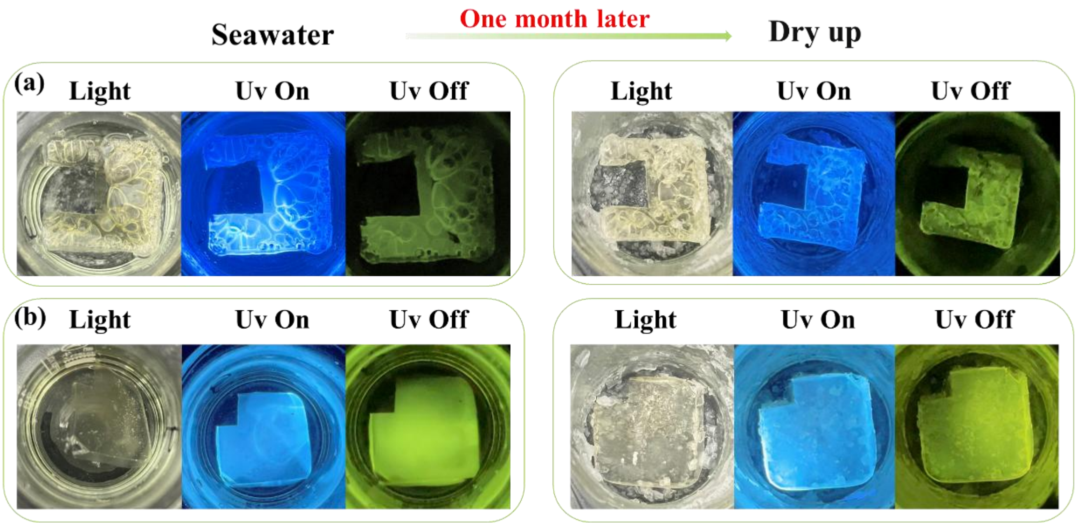

Supplementary Figure. 52 Long-Term Seawater Exposure and Afterglow Retention of Ph-Bd[g] Films. Photographs of Ph-Bd[g]@PMMA (a) and Ph-Bd[g]@PVB (b) samples immediately after immersion in seawater and after one month of seawater exposure followed by evaporation-induced solid residue formation, captured under daylight, UV excitation (UV on), and after UV excitation is turned off (UV off).

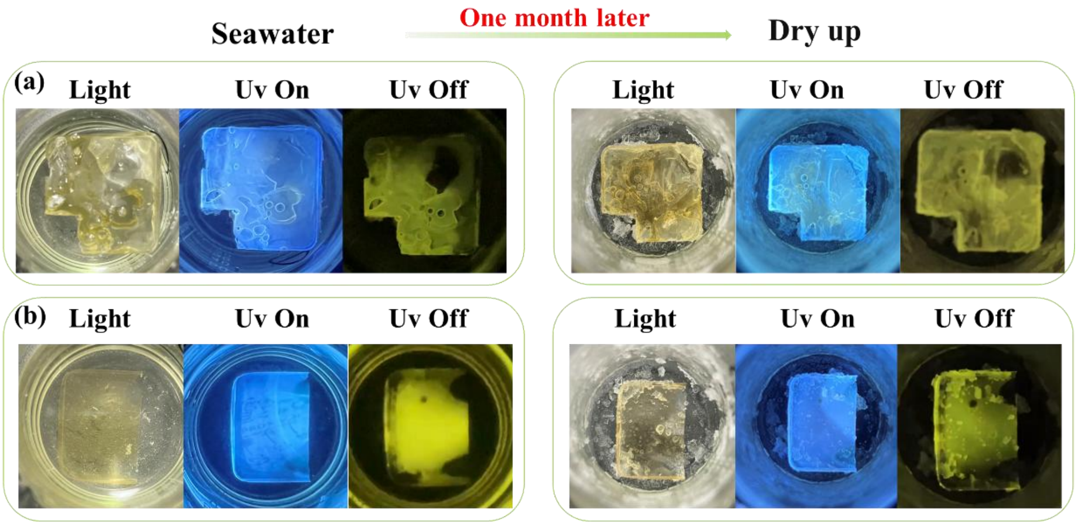

Supplementary Figure. 53 Long-Term Seawater Exposure and Afterglow Retention of Ph-Bd[e] Films. Photographs of Ph-Bd[e]@PMMA (a) and Ph-Bd[e]@PVB (b) samples immediately after immersion in seawater and after one month of seawater exposure followed by evaporation-induced solid residue formation, captured under daylight, UV excitation (UV on), and after UV excitation is turned off (UV off).

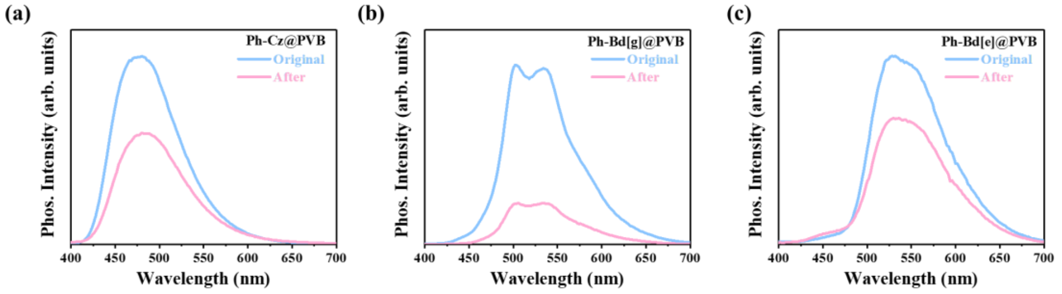

Supplementary Figure. 54 Seawater-Immersion Stability of Phosphorescence in PVB-Doped Films. Phosphorescence emission spectra of Ph-Cz@PVB (a), Ph-Bd[g]@PVB (b), and Ph-Bd[e]@PVB (c) films recorded before and after seawater immersion.

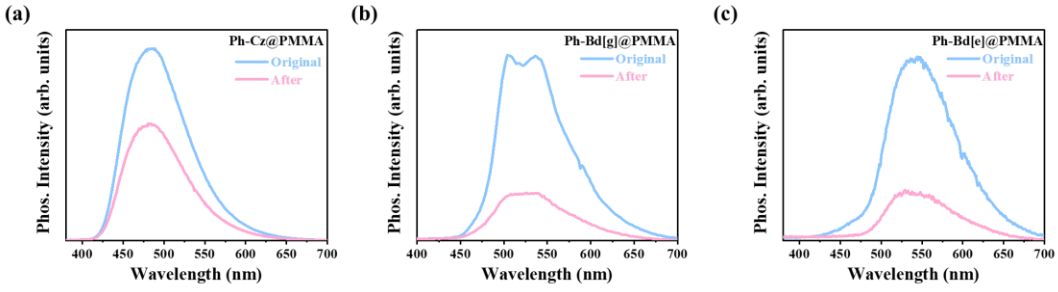

Supplementary Figure. 55 Seawater-Immersion Stability of Phosphorescence in PMMA-Doped Films. Phosphorescence emission spectra of Ph-Cz@PMMA (a), Ph-Bd[g]@PMMA (b), and Ph-Bd[e]@PMMA (c) films recorded before and after seawater immersion.

Supplementary Table 6. Matrix-Universal RTP of Ph-Cz and Ph-Bd Isomers. RTP performance of Ph-Cz, Ph-Bd[g], Ph-Bd[e], and Ph-Bd[f] doped systems utilized in matrix universality.

Molecule	matrix	RTP color	T_1 (eV)	τ_P (s)	Φ_P (%)
Ph-Cz	PVA	Blue	2.70	4.23	16.6
Ph-Bd[g]	PVA	Green	2.29	0.93	13.4
Ph-Bd[e]	PVA	Yellow	2.05	0.74	12.9
Ph-Bd[f]	PVA	Red	1.65	0.20	10.7
Ph-Cz	PVP	Blue	2.54	0.63	16.2
Ph-Bd[g]	PVP	Green	2.26	0.56	13.7

Ph-Bd[e]	PVP	Yellow	2.11	0.40	13.1
Ph-Bd[f]	PVP	-	1.65	-	-
Ph-Cz	PVB	Blue	2.68	0.91	9.2
Ph-Bd[g]	PVB	Green	2.43	0.38	11.3
Ph-Bd[e]	PVB	Yellow	2.01	0.26	10.9
Ph-Bd[f]	PVB	-	1.69	-	-
Ph-Cz	PMMA	Blue	3.60	0.41	7.1
Ph-Bd[g]	PMMA	Green	2.23	0.25	9.7
Ph-Bd[e]	PMMA	Yellow	2.05	0.17	8.6
Ph-Bd[f]	PMMA	-	1.65	-	-

References

1. Kim, H. *et al.* Efficient synthesis of aryl-substituted carbazoles via tandem double or triple suzuki coupling and cadogan cyclization. *Tetrahedron* **73**, 1413-1423 (2017).

2. Porcheddu, A. *et al.* Mechanochemical Fischer indolisation: an eco-friendly design for a timeless reaction. *Green Chem.* **24**, 4859-4869 (2022).

3. Kim, J. *et al.* A Unified Approach to Mono- and 2,3-Disubstituted <i>N</i> -H Indoles. *Synlett* **34**, 1719-1722 (2023).

4. Manikandan, R. *et al.* Ruthenium-Catalyzed Hydroarylation of Anilides with Alkynes: An Efficient Route to Ortho-Alkenylated Anilines. *Org. Lett.* **16**, 912-915 (2014).

5. Cheng, H. *et al.* Mechanochemical Ruthenium-Catalyzed Hydroarylations of Alkynes under Ball-Milling Conditions. *Org. Lett.* **19**, 6284-6287 (2017).

6. Andries-Ulmer, A. *et al.* Fluorine as a Traceless Directing Group for the Regiodivergent Synthesis of Indoles and Tryptophans. *J. Am. Chem. Soc.* **140**, 13034-13041 (2018).

7. Li, Y. *et al.* Metal-Free Synthesis of Indole via NIS-Mediated Cascade C-N Bond Formation/Aromatization. *J. Org. Chem.* **80**, 3841-3851 (2015).
