# Supplementary material for: Isomer design unlocks rainbow phosphorescence
Source: Nat Commun. 2026 Mar 17;17:4093. doi: 10.1038/s41467-026-70784-7 (PMC13144461; doi:10.1038/s41467-026-70784-7)
Supplement: Supplementary file 2 — Description of Additional Supplementary Files [file 41467_2026_70784_MOESM2_ESM.pdf]

Description of Additional Supplementary Files

File Name: Supplementary Data 1

Description: Cartesian coordinates of compounds Ph-Cz, Ph-Bd[g], Ph-Bd[e], and Ph-Bd[f].
